# A diet rich in C_3_ plants reveals the sensitivity of an alpine mammal to climate change

**DOI:** 10.1111/mec.14842

**Published:** 2018-09-17

**Authors:** Sabuj Bhattacharyya, Deborah A. Dawson, Helen Hipperson, Farah Ishtiaq

**Affiliations:** ^1^ Centre for Ecological Sciences Indian Institute of Science Bangalore India; ^2^ Department of Animal and Plant Sciences Western Bank University of Sheffield Sheffield UK

**Keywords:** Alpine mammal, C_3_ photosynthetic pathway, climate change, diet, herbivore, Himalaya, metabarcoding

## Abstract

Plant–herbivore interactions provide critical insights into the mechanisms that govern the spatiotemporal distributions of organisms. These interactions are crucial to understanding the impacts of climate change, which are likely to have an effect on the population dynamics of alpine herbivores. The Royle's pika (*Ochotona roylei*, hereafter pika) is a lagomorph found in the western Himalaya and is dependent on alpine plants that are at risk from climate change. As the main prey of many carnivores in the region, the pika plays a crucial role in trophic interactions. We examined topographical features, plant genera presence and seasonal dynamics as drivers of the plant richness in the pika's diet across an elevational gradient (2,600–4,450 m). We identified 79 plant genera in the faecal pellets of pikas, of which 89% were forbs, >60% were endemic to the Himalaya, and 97.5% of the diet plant genera identified followed the C_3_ photosynthetic pathway. We found that, during the premonsoon season, the number of genera in the pika's diet decreased with increasing elevation. We demonstrate that a large area of talus supports greater plant diversity and, not surprisingly, results in higher species richness in the pika's diet. However, in talus habitat with deep crevices, pikas consumed fewer plant genera suggesting they may be foraging suboptimally due to predation risk. The continued increase in global temperature is expected to have an effect on the distribution dynamics of C_3_ plants and consequently influence pika diet and distribution, resulting in a significant negative cascading effect on the Himalayan ecosystem.

## 1 INTRODUCTION

Plant–herbivore interactions provide critical insights into diverse ecological processes shaping community dynamics across an array of fields linking co‐evolution (Ehrlich & Raven, 1964; Johnson, Campbell, & Barrett, 2015), chemical ecology (Hay & Fenical, 1988; Rasher et al., 2015), foraging and nutritional ecology (Raubenheimer, Simpson, & Mayntz, 2009; Wetzel, Kharouba, Robinson, Holyoak, & Karban, 2016). These interactions are key determinants of the mechanisms which govern a species’ spatial distribution, abundance, community dynamics, primary productivity and function in the food web (Lurgi, López, & Montoya, 2012; Oerke, 2006) and the impact of changing climate on landscape‐level patterns in a terrestrial ecosystem (Mulder, Koricheva, Huss‐Danell, Hogberg, & Joshi, 1999). Determining the diet of a herbivore is fundamental to understanding trophic interactions and assessing dietary plasticity to climate change, and for developing effective monitoring, management and conservation strategies (Bernstein et al., 2007).

In alpine ecosystems, pikas (*Ochotona* spp.), small‐bodied lagomorphs, are good examples of how a changing climate impacts plant–herbivore interactions. The American pika (*Ochotona princeps*) is a generalist herbivore whose diet relies heavily on alpine plants (Ray, Beever, & Loarie, 2012). However, climate‐induced changes in vegetation distribution and composition (Erb, Ray, & Guralnick, 2011; Jeffress, Rodhouse, Ray, Wolff, & Epps, 2013; Rodhouse et al., 2010; Wilkening, Ray, Beever, & Brussard, 2011) led to historic extinctions of local populations and recent range contraction (Beever, Brussard, & Berger, 2003; Beever, Ray, Mote, & Wilkening, 2010; Stewart, Wright, & Heckman, 2017). During the late Miocene, the reduction in the availability of preferred C_3_ diet plants was linked to the extinction of many pika genera in various parts of Africa, Eurasia and North America (Ge et al., 2012, 2013).

The Royle's pika (*Ochotona roylei*) is a widely distributed alpine mammal found in rocky areas at elevations ranging from 2,400 to 5,000 m in the Himalayan region. It is found on rock talus and is an obligate herbivore species with limited dispersal ability due to its small body size and fairly narrow ecological niches (Bhattacharyya, Adhikari, & Rawat, 2010; Bhattacharyya, 2013; Bhattacharyya, Adhikari, & Rawat, 2014a,b; Bhattacharyya, Dutta, Adhikari, & Rawat, 2015; Bhattacharyya & Smith, 2018). The Royle's pika is a diurnal species and produces distinct piles of faecal droppings (Bhattacharyya et al., 2014a,b). Similar to other lagomorphs in the Himalayan region, the Royle's pika does not hibernate but forages throughout the year on alpine plants along the talus–vegetation interface (Bhattacharyya et al. 2010; Bhattacharyya, 2013; Bhattacharyya et al., 2014a,b, 2015; Bhattacharyya & Smith, 2018). Hence, unlike other *Ochotona* species, the Royle's pika does not store hay for winter survival (Bhattacharyya & Smith, 2018). Previous dietary analysis using visual observations indicated that forbs and grasses were the preferred plants in the pikas diet (Awan, Minhas, Ahmed, & Dar, 2004; Bhattacharyya, Adhikari, & Rawat, 2013; Shrestha, Khanal, & Karki, 1999), which have a significantly higher protein, lipid and moisture content (Bhattacharyya et al., 2013; Ge et al., 2012). Pikas are able to consume plants with high toxic components including secondary metabolites (Bhattacharyya et al., 2013), which are usually avoided by large herbivorous mammals (Sorensen, McLister, & Dearing, 2005). In addition, the Royle's pika is the main prey for a range of carnivore species, for example*,* yellow‐throated marten (*Martes flavigula*), Himalayan weasel (*Mustela sibirica*), snow leopard (*Panthera uncia*) and red fox (*Vulpes vulpes*), in alpine and subalpine ecosystems in the Himalayan region (Oli, Taylor, & Rogers, 1994; Roberts, 1977). Thus, pikas play a crucial role in plant–herbivore trophic interactions, and their extinction or range contraction is likely to have a significant negative cascading effect on the functioning of the whole ecosystem.

DNA metabarcoding using high‐throughput sequencing enables the identification of dietary species using DNA extracted from faecal samples more accurately than traditional visual and microscopic observation of faecal samples (Kartzinel et al., 2015). This technique has been used to identify herbivore gut contents, revealing cryptic functional diversity and niche partitioning (Kress, García‐Robledo, Uriarte, & Erickson, 2015). Plastid genes such as *rbc*L (Group et al., 2009) and nuclear ribosomal internal transcribed spacer (ITS; Hollingsworth, 2011) are commonly used for plant metabarcoding (Hollingsworth, 2011). We aimed to analyse faecal pellets using metabarcoding to identify the plants in the diet of the Royle's pika and examine the effects of talus characteristics, topography and plant richness; abundance; and seasonal dynamics (pre‐ and postmonsoon) on diet, across five sites in the western Himalaya, India. Food availability (plant species’ presence and abundance; Huntly, Smith, & Ivins, 1986; Dearing, 1995, 1996; Wilkening et al., 2011; Bhattacharyya et al., 2013, 2014a; Bhattacharyya & Ray, 2015), habitat topography (elevation, slope, aspect; Walker, Halfpenny, Walker, & Wessman, 1993; Deems, Birkeland, & Hansen, 2002; Wilkening et al., 2011; Rodhouse et al., 2010; Gurung et al., 2017) and predation risk (rock cover, crevice depth, distance to the nearest area of talus; Calkins, Beever, Boykin, Frey, & Andersen, 2012; Bhattacharyya et al., 2014a,b; Castillo, Epps, Davis, & Cushman, 2014; Bhattacharyya et al., 2015) significantly impact the foraging ecology of the Royle's pika and potentially influence access to nutritive plants, and thereby affect individual fitness (Bhattacharyya, 2013). For talus‐dwelling *Ochotona* spp., talus size (area) and connectivity between talus are known to influence the habitat occupancy (Franken & Hik, 2004). In Royle's pikas, a high proportion of rock cover in talus habitat provides refuge from predation risk, which in turn increases their habitat occupancy and abundance (Bhattacharyya et al., 2014a, 2015). Topographical features determine the distribution and abundance of alpine plants (Bruun et al., 2006). The prehistoric distribution of small mammals (Ochotonidae), highly adapted to arctic or alpine environment, was closely associated with preferred C_3_ food plant diet which is high in protein and moisture content (Ge et al., 2012). Whilst the interplay between plants and herbivores is a key determinant of community structure (see Dolezal et al., 2016; Wisz et al., 2013), climate‐induced expansion of C_4_ plants is believed to have resulted in extinction and range contraction in pikas (Ge et al., 2012). Furthermore, in highly seasonal montane habitats, dietary constraints in herbivores tend to be strongly linked to quality of forage available. In the Himalayan region, both plant quality and available biomass may act as constraints for pikas. Seasonal dynamics in diet selection can reflect dietary adaptations (plasticity) in a seasonal alpine habitat. Our Royle's pika diet analysis will provide insights into the plant genera selected during foraging and test the following hypotheses:

1. Seasonal difference in diet (premonsoon versus postmonsoon) will demonstrate dietary flexibility, and the pikas will select plants with the highest nutrient gain possible from the feeding habitats available.
2. Larger areas of talus habitat will result in more plant availability and will increase species richness of the diet.
3. Talus characteristics such as rock cover and depth of crevices would increase species richness in the diet by providing refuge from predators and allowing pikas to access larger foraging ground.
4. Forbs and grasses constitute the largest proportion of their diet.
5. Pikas prefer diets rich in C_3_ plants, which signify potential threat due to climate change as C_3_ plants are dependent on high rainfall and low temperature.

## 2 MATERIALS AND METHODS

### 2.1 Study area, collection of faecal pellets and habitat data

**Figure 1:**
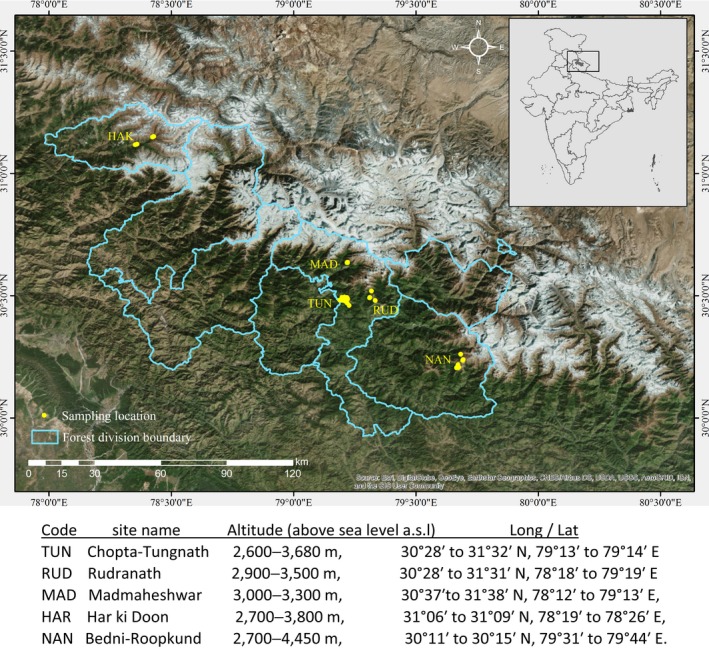
Faecal pellet sampling sites in Garhwal region of Uttarakhand, India [Colour figure can be viewed at wileyonlinelibrary.com]

The Royle's pika inhabits rock crevices in talus fields with a home range of approximately 50 m^2^ (Bhattacharyya et al., 2015; Kawamichi, 1968). During 2014–2015, 104 rock talus habitats were surveyed and faecal pellets collected from five sites at elevations ranging between 2,600 and 4,450 m above sea level (a.s.l.): Chopta‐Tungnath (TUN), Rudranath (RUD), Madmaheshwar (MAD: Kedarnath Wildlife Sanctuary), Har ki Doon (HAR: Govind Wildlife Sanctuary) and Bedni‐Roopkund (NAN: Nandadevi Biosphere Reserve; Figure 1) in Garhwal, Uttarakhand, India. The sampling was conducted in two seasons: postmonsoon (October to November 2014) in TUN, RUD and HAK and premonsoon (May to June 2015) in TUN, MAD and NAN.

Following Bhattacharyya et al. (2015), we selected 50 m^2^ survey plots in each talus habitat, and these were searched for fresh piles of faecal pellets (moist, dark brown/black). One site (HAK) had only old (>2 months) faecal piles (light brown and dry). Faecal pellets (TUN premonsoon = 42, postmonsoon = 40; NAN = 43; MAD = 19; HAK = 20; RUD = 8) were collected in airtight 2‐ml plastic tubes containing silica gel (Merck, India) and were labelled with date, sampling location and geographic coordinates (latitude and longitude). In addition, the total talus area (in m^2^) around each survey plot, distance from nearest talus (intertalus distance, in m), depth of deepest crevices in the talus (<0.5 m, 0.5–1 m, 1–1.5 m and >1.5 m) and topographical features such as elevation, slope and aspect were recorded. For each 50 m^2^ plot, we visually estimated the proportion of rock cover, forbs (e.g., *Geum elatum* and *Potentilla atrosanguinea*), grasses (e.g., *Danthonia sp*. and *Poa sp*.), shrubs (e.g., *Rhododendron campanulatum and/or Vivernum sp*.) and trees (e.g., *Abies pindrow, A. spectabilis, Quercus semecarpifolia* and *Rhododendron arboretum*; Van Hees & Mead, 2000; Bhattacharyya et al., 2015).

### 2.2 DNA metabarcoding of faecal pellets

The Royle's pika's diet is rich in secondary metabolites (Bhattacharyya et al., 2013), which often inhibit downstream enzymatic reactions in polymerase chain reaction (PCR; Weishing, Nybom, Wolff, & Meyer, 1995). Therefore, we homogenized 20–30 mg of the faecal samples and extracted DNA using QiAamp DNA Stool Kit (Qiagen, Manchester, UK) following minor modifications in the manufacturer's protocol (e.g., overnight incubation at 56°C with ASL stool lysis buffer; Qiagen Inc., Germany). As Royle's pika were the only lagomorph species present in the study area (Bhattacharyya, 2013; Green, 1985) with very distinct faecal pellets, the chances of misidentification of the faecal samples were ruled out.

We amplified the ITS2 region of plant nuclear DNA using primer pair UniPlantF (5′‐TGTGAATTGCARRATYCMG‐3′) and UniPlantR (5′‐CCCGHYTGAYYTGRGGTCDC‐3′; 187–380 bp; Moorhouse‐Gann et al., 2018) and the *rbcL* region of chloroplast DNA using primer pair *rbcL*a‐F (5′ ATGTCACCACAAACAGAGACTAAAGC‐3′ and *rbcL*a‐R (5′‐GTAAAATCAAGTCCACCRCG‐3′; 553 bp; Levin et al., 2003; Kress et al., 2009; Yoccoz et al., 2012). Primers had overhang adapter sequences added to the 5′ end for the initial PCR amplification, following Campbell, Harmon, and Narum (2015). This allows unique 6 bp dual index sequences to be added along with Illumina capture sequences to each sample in a subsequent PCR step prior to pooling samples for sequencing. Initial PCR amplifications were performed in 10 μL reaction volumes including 5 μL of Qiagen Multiplex PCR Master Mix (Qiagen, Manchester, UK), 1 μL of each primer (1 μM), 2 μL of nuclease‐free water (Thermo Fisher Scientific, Inc.) and 1 μL of DNA template. Reaction conditions were as follows: initial denaturation at 95°C for 15 min; 44 cycles of 94°C for 90 s, 55°C for 30 s and 72°C for 60 s; and final extension of 72°C for 10 min. We used DNA from Quaking grass (*Briza media*) as positive control and included a negative control for each PCR run (Zarzoso‐Lacoste, Corse, & Vidal, 2013). The PCR products amplified by each primer pair were separated on a 1% agarose gel stained with SYBR^®^ Safe, amplicon size was compared to a 100 bp ladder (Thermo Fisher Scientific, Paisley, UK), and amplification success was assessed. In addition, each PCR product was quantified using a BioAnalyzer (Agilent Technologies, Santa Clara, CA) to accurately estimate the amplicon size and DNA concentration. Only amplicons with clear visible band following electrophoresis were processed further. The ITS2 and *rbcL* PCR products were pooled in equal amounts and quantified using a Qubit (Thermo Fisher Scientific, Waltham, MA) to ensure approximately equal amounts of amplicon DNA were used in the second amplification step to attach the Illumina tags. This PCR amplification was performed in a 10 μL reaction volume including 5 μL of Qiagen Multiplex PCR Master Mix (Qiagen, Manchester, UK), 0.5 μL of forward and reverse Illumina Multiplex Identifier (MID) tagged or indexed primers (1 μM), 2 μL DNase and nuclease‐free water (Thermo Fisher Scientific, Inc.) and 4 μL of DNA template (pika faecal sample or pooled PCR product from previous ITS2 and *rbcL* amplification). Reaction conditions were as follows: initial denaturation at 95°C for 15 min; 10 cycles of 98°C for 10 s, 65°C for 30 s and 72°C for 30 s; and final extension of 72°C for 5 min. The concentrations of these PCR products (2 μL) were measured using a fluorimeter (Thermo Fisher Scientific, Paisley, UK) and pooled together in batches of eight samples with similar concentrations. Each batch of pooled samples was then purified following the Agencourt AMPure XP PCR Purification Kit protocol (Beckman Coulter Genomics, Aus.) to remove nontarget DNA fragments and primer dimer and eluted in 40 μL of ultrapure water (Murray, Coghlan, & Bunce, 2015). Each cleaned pool was then analysed on a Tape Station (Agilent Technologies, USA) to check for successful removal of primer dimer. Each pool was then serially diluted and quantified using quantitative PCR (Applied Biosystems, CA, USA) where a diluted custom synthetic oligonucleotide of known molarity was used as standard to determine the final volume of library to use for sequencing. The qPCR amplification was performed in a 25 μL volume containing KAPPA library quantification kit (Kappa Biosystem, Inc.), 0.4 μM of specific forward and reverse primer and 2 μL of pooled amplicon library and using these reaction conditions: 95°C for 5 min, followed by 35 cycles of 95°C for 30 s and 60°C for 45 s. Based on the cycle threshold (CT) values observed, each pool of eight samples were combined in equimolar proportions (4 nM) to make a single library with unique Illumina tagged PCR product for all 124 samples to run using the 500 cycle v2 (2 × 250 bp paired‐end reads) sequencing kit on the MiSeq Desktop Sequencer (Illumina, San Diego, CA, USA).

### 2.3 Identification of plant diet from sequencing data

The bioinformatic analyses were performed using Iceberg, the High Performance Computing Cluster at the University of Sheffield, UK. The paired‐end reads were filtered for quality (minimum quality score 20 over a 4 bp sliding window) and any Illumina adapter sequences removed using Trimmomatic v 0.32 (Bolger, Lohse, & Usadel, 2014), retaining only reads of at least 90 bp in length. For ITS2, filtered sequences were then aligned using FLASH (Magoč & Salzberg, 2011), aligned sequences with matches to the ITS2 primer sequences only were extracted, and primer sequences were removed using the “trim_seqs” command in Mothur (Schloss et al., 2009). The ITS2 region was extracted from the whole amplicon sequence (which includes ~73 bp of 5.8S rDNA sequence, Moorhouse‐Gann et al., 2018) using ITSx (Bengtsson‐Palme et al., 2013). The “derep_fulllength” and “uchime2_denovo” commands were used in usearch software v 9.2.64 (Edgar, 2010) to eliminate all sequences which had less than 10 copies per sample and any chimeric sequences. Each unique ITS2 sequence found in the data set was then compared against the NCBI GenBank nucleotide database using the BLAST algorithm (Altschul et al., 1997) to assign a taxonomic unit for each plant sequenced identified. Only matches with at least a 97% identity to the reference sequence were retained for downstream analysis. The software metagenome analyzer v 4 (MEGAN, Huson et al., 2016) was used against NCBI taxonomic framework for mapping and visualization of the BLAST results, keeping all default parameters for the LCA assignment algorithm except the bit score minimum support threshold, which was set at 1%. For *rbcL*, the analysis pipeline differed slightly as we did not expect a region of overlap between the paired sequencing reads with these ~550 bp long amplicons. Instead, we used a custom script to reverse complement read 2 and combine with read 1 to form an *rbcL* sequence starting and ending with the primer sequences but having at least a 50 bp gap in the middle. Prior to performing the BLAST search, we also clustered the dereplicated and nonchimeric at 97% sequence similarity using the “cluster_fast” commands in usearch. Owing to limited reference plant database from the Himalayan region, we were able to identify plants only at genus level. The reliability of each molecular operational taxonomic unit (mOTU) corresponding to a specific plant genus was further evaluated against a published list of plants reported from the study area (Bhattacharya, Sathyakumar, & Rawat, 2007; Rai, Adhikari, & Rawat, 2012), and online plant [e.g., flora Himalaya database (http://www.leca.univ-savoie.fr/db/florhy/infos.html), Dobremez et al., [Link]] and biodiversity databases [e.g., The Global Biodiversity Information Facility (https://www.gbif.org/)], which provided information on elevation and geographic range of the plant genus. Plant genera not reported from the Himalayan region were not considered for further analysis.

### 2.4 Information of ecological and evolutionary linkages of plants in Royle's pika diet

The adaptation ability, ecological requirements, physiology and nutritional quality of plants often depend on their photosynthetic pathways (C_3_, C_4_ or Crassulacean acid metabolism [CAM]; Ehleringer & Monson, 1993). The photosynthetic pathway of plants belonging to the same genus is often considered the same (Osborne et al., 2014; Sage, 2016). Therefore, we obtained information on photosynthetic pathways of pika food plants from published sources (Ge et al., 2012; Osborne et al., 2014; Bhattacharyya & Ray,^2015^; Sage, 2016), and information on evolutionary origin (e.g., endemic to Himalaya, Tropical and Holarctic) average upper and lower elevation distribution range of each diet plant genus was obtained from flora of Himalaya database (http://www.leca.univ-savoie.fr/db/florhy/infos.html; Dobremez et al. [Link]), Global Biodiversity Information Facility (https://www.gbif.org/search) and IUCN red list of threatened species online portal (http://www.iucnredlist.org/).

### 2.5 Statistical analysis

#### 2.5.1 Plant composition and seasonal dynamics in diet

We compared the plant diet composition at family and genera level. We used multiple response permutation procedure (MRPP; Mielke, Berry, & Johnson, 1976; Zimmerman, Goetz, & Mielke, 1985) to understand seasonal variation in diet composition at plant genera level across multiple sites. We used contingency table analysis to test for heterogeneity in plant family in diet across sites, which was assessed by G tests followed by partitioned analyses (Sokal & Rohlf, 1995). In addition, we have used generalized linear models (GLMs) to understand the effect of elevation on plant diet richness (number of genera) across seasons and comparison of plant families across sites sampled in the same season. The significance of fixed effects was evaluated with Wald's chi‐square tests (Bolker et al., 2009). To understand whether relative contribution of vegetation type (forbs, grass, shrub and tree) in pika diet is proportional to their availability in the environment, we conducted compositional analysis (Aebischer, Robertson, & Kenward,^1993^) using Adehabitat v 1.8.20 package in R (Calenge, 2006). We restricted our analysis to the vegetation types which qualified within the criteria of a minimum of two data points greater than zero per vegetation category in the environment data set (Calenge, 2006). In addition, the contribution of each plant genus to overall pika diet with their corresponding evolutionary origin and distribution range was visually explored using box plots.

#### 2.5.2 Modelling effect of talus habitat on diet richness

We investigated the effect of food availability (tree, shrub, grass and forbs cover), predation risk (talus area, distance between talus, and depth of crevices) and talus topography (elevation, aspect and slope) on plant diet richness. We fitted 32 GLM models and used AIC‐based multimodel inference to identify well‐supported statistical models that describe the relationships between plant diet richness and biological parameters relevant to foraging ecology of Royle's pika (Table S1). Season was not incorporated as an explanatory variable in the model as not all sites were surveyed in both seasons. Model averaging was conducted using the MuMin v package in R (Barton & Barton, 2018) on the model set generated from the global model, applying a threshold‐corrected Akaike's information criterion (ΔAICc; Burnham & Anderson, 2002) to select the best candidate model based on the lowest (AIC) values corrected for sample size bias or AICc values (ΔAICc) > 2 units than the quality of other competing models. Alternatively, when two or more models had difference in AICc < 2, we used multimodel‐averaged estimates to check the validity of the top ranking model in each case, only including models with ΔAICc < 2 (Burnham & Anderson, 2002). The relative importance (RI) of each parameter after model averaging was calculated by summing Akaike's weight (*wi*) across all models in which the parameter was present. All data were checked for normality and corrected for overdispersion if required. We tested for possible collinearity of the explanatory variables using Pearson's correlation analysis in the global model; the mean correlation was 0.14, and the strongest was 0.64. Correlated variables were used only in interactive models but not in additive models (Graham, 2003). All analyses were conducted in R v 3.3.3 (The R Foundation for Statistical Computing, http://www.r-project.org/).

## 3 RESULTS

The Illumina run produced a total of 3,685,142 paired‐end reads (2 × 250 bp), with an average of 29,719 reads per sample (range 172–113,816 reads, *n* = 124).

### 3.1 Plant composition and seasonal dynamics in pika diet

We successfully obtained information from 110 faecal pellets (after controlling for data quality) representing 66 out of 104 sampled rocky talus plots. A total of 79 plant genera (ITS2 = 54, *rbcL* =13, 12 were common to both) were identified. We retrieved 62 genera and 28 families of forbs, ten genera and one family of grass, three genera and three families of shrubs, and three genera and three families of trees (Table S2).

Of the 32 plant families recorded in the pikas’ diet, Asteraceae, Poaceae, Primulaceae, Ranunculaceae, Rosaceae and Scrophulariaceae showed significant differences across sites in the mean proportion of occurrence within diet (Table 1). We found no significant difference in proportion of plant families in the premonsoon (GLM: *F*_12_ = 111.42, *p* < 0.25) and postmonsoon season (GLM: *F*_6_ = 49.74, *p *<* *0.08).

**Table 1:** Presence of plant families in the Royle's pika's diet across each sampled site (TUN: Tungnath; MAD: Madmaheshwar; NAN: Bedni‐Roopkund; RUD: Rudranath; and HAK: Har ki Doon), with results from G test (G statistics and *p* values), to estimate differences in the mean proportion of plant families within diet. Proportion of plant family is presented for each site; those highlighted in bold differ across sites at *p *<* *0.05

Family	Total genus	Premonsoon			Postmonsoon			G	*p*
		TUN (*n* = 27)	MAD (*n* = 13)	NAN (*n* = 32)	TUN (*n* = 26)	RUD (*n* = 4)	HAK (*n* = 8)		
Apiaceae	5	3.70	0.00	6.25	11.54	25.00	0.00	1.74	0.41
Asteraceae	7	55.50	23.07	15.60	61.50	100.00	12.50	76.41	**<0.0001**
Balsaminaceae^a^	1	0.00	7.69	0.00	0.00	0.00	0.00	a	
Berberidaceae^a^	1	0.00	0.00	6.25	0.00	25.00	0.00	a	
Betulaceae^a^	1	3.70	0.00	0.00	0.00	0.00	0.00	a	
Boraginaceae^a^	2	0.00	7.69	0.00	0.00	0.00	0.00	a	
Brassicaceae^a^	2	0.00	0.00	3.13	0.00	0.00	0.00	a	
Campanulaceae^a^	1	0.00	7.69	0.00	3.85	0.00	0.00	a	
Caprifoliaceae	2	11.11	0.00	3.13	7.69	25.00	0.00	2.69	0.26
Caryophyllaceae	4	14.81	15.38	15.63	18.52	15.38	0.00	4.54	0.47
Crassulaceae^a^	2	0.00	7.69	0.00	0.00	0.00	0.00	a	
Cyperaceae	1	11.11	23.08	9.38	14.81	50.00	0.00	6.9	0.22
Ericaceae	4	18.52	7.69	0.00	26.92	25.00	0.00	2.94	0.7
Fagaceae^a^	1	3.70	0.00	0.00	0.00	0.00	0.00	a	
Gentianaceae^a^	2	3.70	0.00	0.00	0.00	0.00	0.00	a	
Geraniaceae^a^	1	3.70	0.00	0.00	3.85	0.00	0.00	a	
Hypericaceae^a^	1	0.00	0.00	0.00	50.00	0.00	0.00	a	
Lamiaceae^a^	1	0.00	0.00	0.00	3.85	0.00	0.00	a	
Onagraceae	3	18.52	23.08	6.25	46.15	0.00	0.00	3.14	0.2
Papilionaceae^a^	2	11.11	0.00	0.00	0.00	0.00	0.00	a	
Poaceae	10	96.26	100.00	25.00	100.00	100.00	25.00	179.23	**<0.0001**
Polygonaceae	4	0.00	30.77	6.25	3.85	0.00	0.00	2.32	0.31
Primulaceae	2	51.85	98.27	34.38	22.22	7.69	3.13	13.93	**<0.001**
Ranunculaceae	4	59.26	92.31	9.38	30.77	25.00	12.50	41.89	**<0.0001**
Rosaceae	6	96.30	92.31	56.25	92.31	100.00	25.00	210.07	**<0.0001**
Rubiaceae	1	3.70	0.00	6.25	0.00	0.00	0.00	1.4	0.49
Salicaceae^a^	1	0.00	0.00	0.00	3.85	0.00	0.00	a	
Saxifragaceae	2	7.41	7.69	3.13	11.54	0.00	0.00	1.78	0.41
Scrophulariaceae	2	25.93	30.77	9.38	23.08	50.00	0.00	18.7	**<0.002**
Orobanchaceae	1	18.52	15.38	3.13	3.85	0.00	0.00	4.2	0.11
Urticaceae^a^	1	0.00	0.00	3.13	3.85	0.00	0.00	a	
Violaceae^a^	1	0.00	0.00	3.13	3.00	0.00	0.00	a	

Note

a Plant family not considered for comparison across sites as they were found only in one study site (in grey shade).

In the premonsoon season, we found significant differences between sites in the proportion of plant family per faecal sample (GLM: *F*_2_ = 126.92, *p *<* *0.0001; proportions listed in Table 1) with NAN having significantly low number of plant families (*t* = −4.18, *p *<* *0.001), whereas TUN showed no difference (*t* = −1.5, *p *<* *0.110). In the postmonsoon season, the proportion of plants in the diet varied significantly across sites (GLM: *F*_2_ = 37.61, *p *<* *0.011) with RUD showing more plant families (*t* = 2.84, *p *<* *0.001) than TUN (*t* = 2.18, *p *<* *0.03). However, high proportion of certain family (e.g., Asteraceae in RUD) in a site is possibly driven by low sample size.

**Figure 2:**
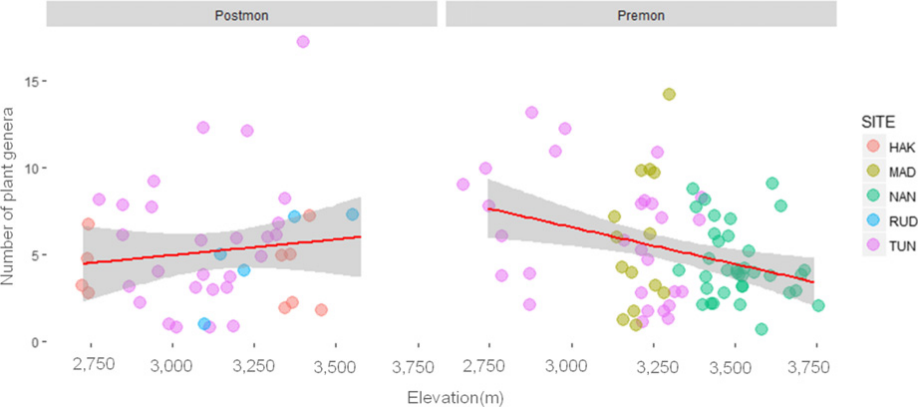
Plant diet richness across an elevation gradient in pre‐monsoon (Premon) and post‐ monsoon season (Postmon) (HAK: Har ki doon; MAD: Madmaheshwar; NAN: Bedni‐roopkund; RUD: Rudranath; TUN: Tungnath; full details of the study sites are provided in Figure 1) [Colour figure can be viewed at wileyonlinelibrary.com]

Forbs constituted 89% of richness, which was significantly higher than both shrubs (7%) and grasses (3%) (GLM: *F*_2_ = 48.91, *p *<* *0.03). We found premonsoon composition of plant genera in diet varied significantly (delta obs. = 2.54, delta exp. = 2.56, A = 0.006, *p *<* *0.05, Figure S1) across sites, whereas there was no variation in plant genera composition in the postmonsoon season. Across seasons within site, Tungnath (TUN) showed significant variation in proportion of plant richness in diet (GLM: *F*_1_ = 18.68, *p *<* *0.0001) with the premonsoon season having lower plant richness (*t* = −4.38, *p *<* *0.0001) than postmonsoon. Plant genus richness during premonsoon varied across elevation, where it increased with a decrease in elevation (Wald's χ^2^ = 9.51, *df *= 1, *p *<* *0.001; Figure 2). Comparing food availability in the environment with dietary results revealed that the Royle's pika prefers forbs (e.g., *Potentila*) over shrubs (e.g., *Viburnum*) in talus habitat during the premonsoon season in TUN (lambda = 0.019, *p *<* *0.01) and NAN (lambda = 0.46, *p *<* *0.01 Figure S2) and during the postmonsoon season in TUN (lambda = 0.11, *p *<* *0.01) and HAK (lambda = 0.007, *p *<* *0.05, Figure S2). No prominent preference for forbs or grasses was observed in MAD (lambda = 0.20, *p *>* *0.05). Overall, composition analysis of vegetation types in the environment and faecal samples across sites during pre‐ and postmonsoon indicated high preference towards forbs (premonsoon: lambda = 0.008, *p *>* *0.01; postmonsoon: lambda = 0.10, *p *>* *0.001). Due to low sample size in RUD, no sitewise vegetation comparison was conducted.

**Figure 3:**
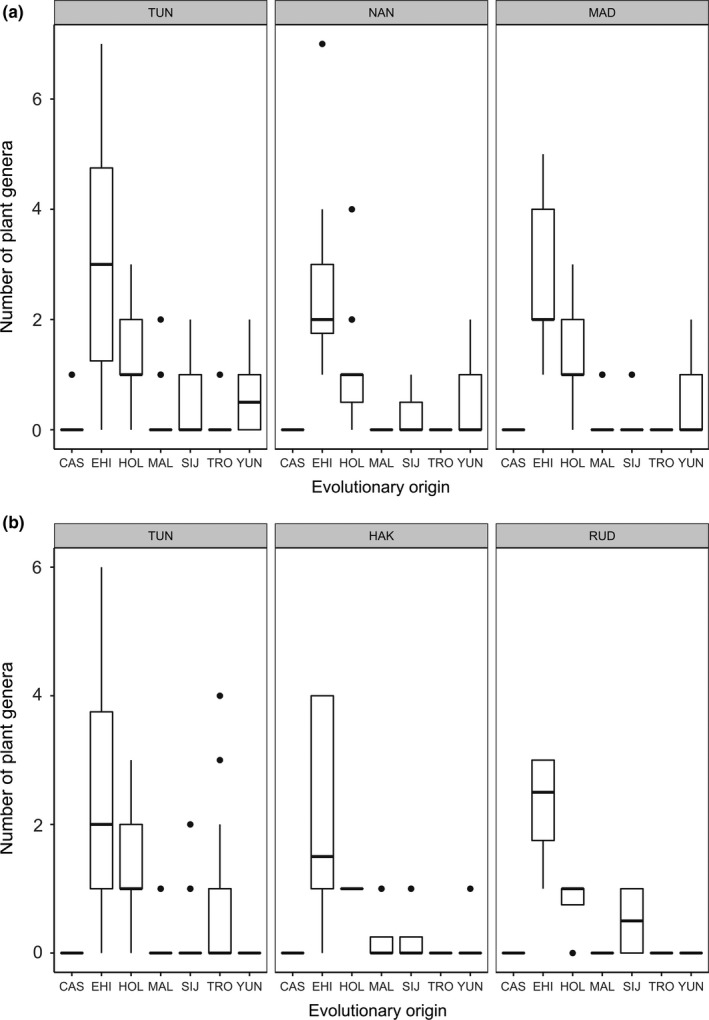
Evolutionary origin of plants per pellet sampled in pre‐monsoon (a) and post‐monsoon (b) diet of Royle's pika in the western Himalaya (CA: Centrasiatic; EH: Himalayan Endemic; HO: Holarctic; MA: South East Asiatic Malaysian; SJ: Sino Japanese or Eastern Asiatic; TR: Tropical; YU: South East Chinese; details of the study sites are provided in Figure 1)

**Figure 4:**
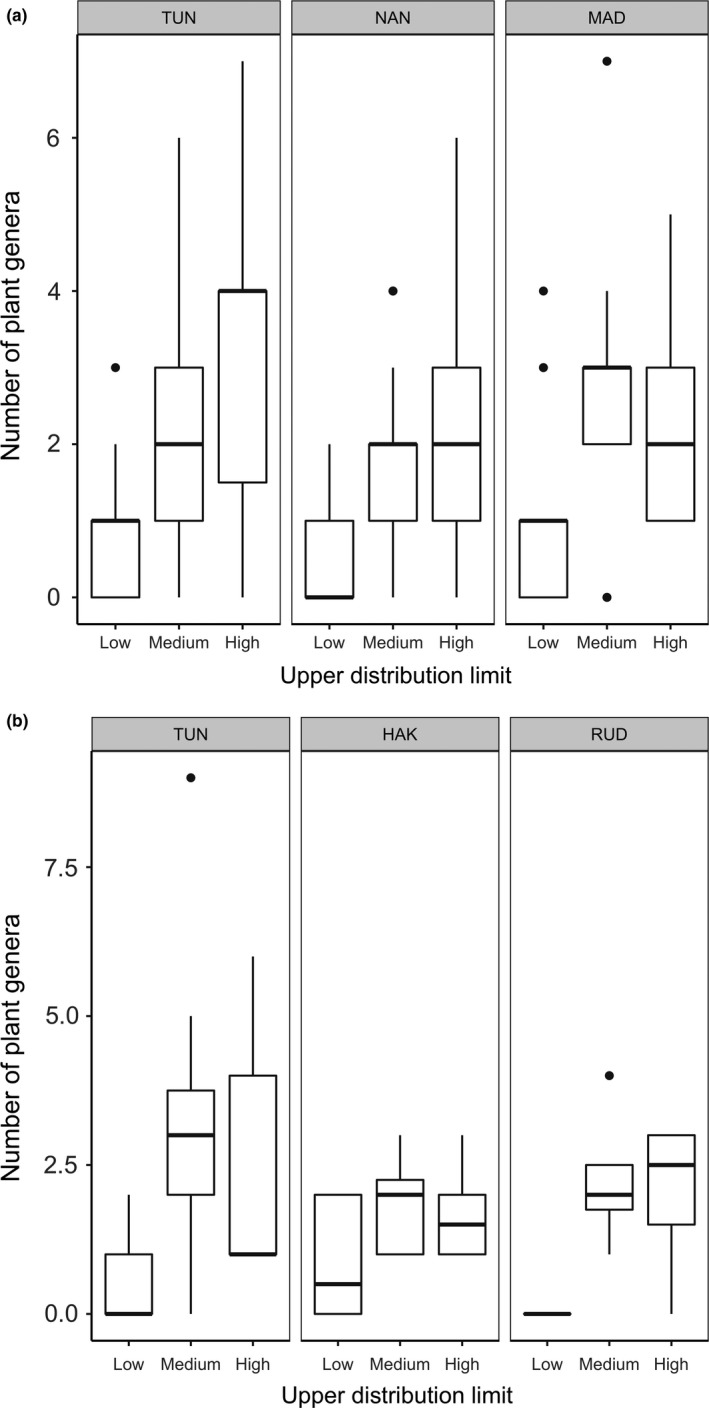
Upper distribution limit of plants detected per sample in pre‐monsoon (a) and post monsoon (b) diet of Royles's pika across elevation (Low = 2,500–3,500 m; Medium = 3,500–4,500 m; High = 4,500–6,000 m; details of the study sites are provided in Figure 1)

**Figure 5:**
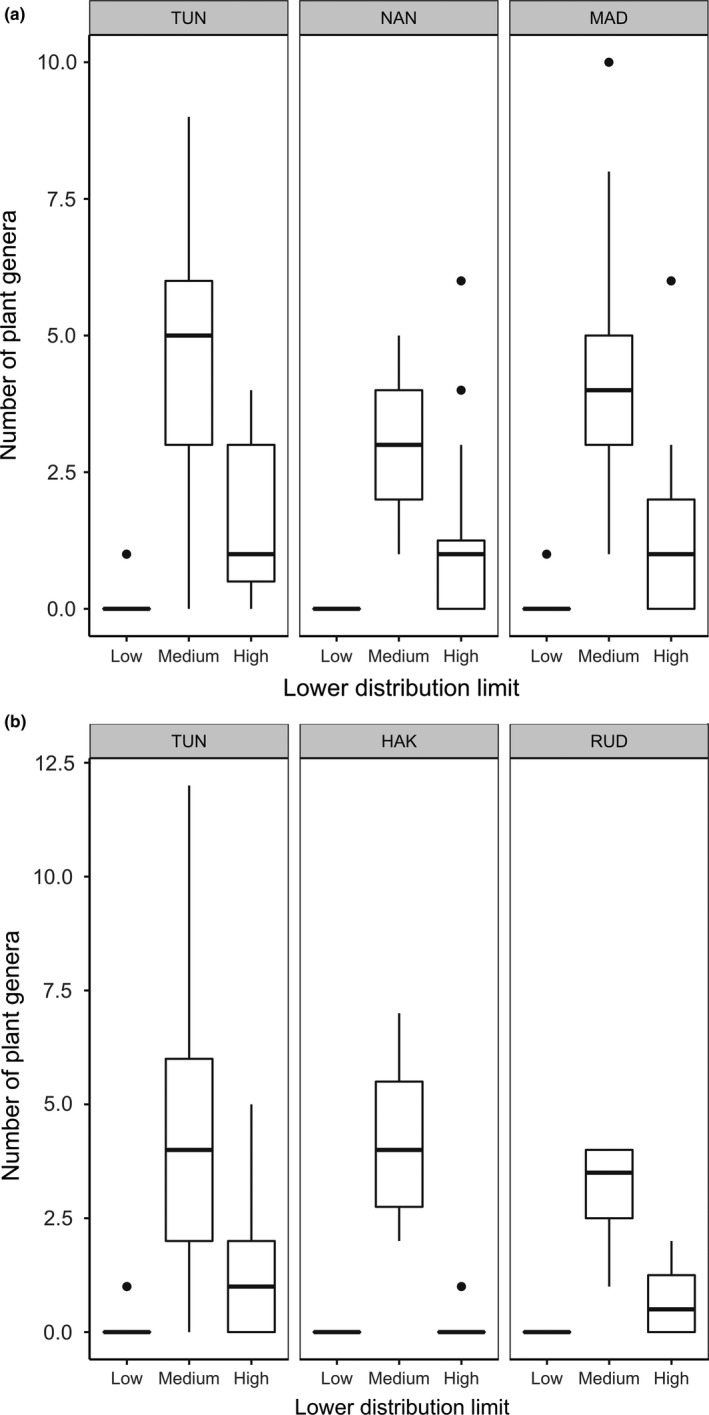
Lower distribution limit of plants detected in pre‐monsoon (a) and post monsoon (b) diet of Royle's pika across elevation (Low = 500–1000 m; Medium = 1,000–2,000 m; High ≥ 2,000 m; details of the study sites are provided in Figure 1)

A high proportion (>50%) of the plant genera (e.g., *Anaphalis* and *Berberis*) detected in both the premonsoon (TUN = 52.02%, MAD = 56.25% and NAN = 56.67%) and postmonsoon (TUN = 53.48%, RUD = 64.28% and HAK = 56.66%) seasons were endemic to the Himalayan region, followed by plants of Holarctic origin (e.g., *Deschampsia*,* Festuca;* premonsoon: TUN = 22.97%, MAD = 28.12% and NAN = 25.0%; postmonsoon: TUN = 26.35%, RUD = 21.42% and HAK = 26.66%) (Figure 3A,B; Table S2). The upper distribution limit of more than 90% of all pre‐ and postmonsoon dietary plants was 3,500 m and above (Figure 4). A significant proportions of premonsoon (TUN = 77.70%, MAD = 75.32% and NAN = 72.8%) and postmonsoon (TUN = 74.49%, RUD = 80.00% and HAK = 97.14%) dietary plants were found to have a lower altitudinal range of 2,000 m to 1,000 m (Figure 5). C_3_ plants constituted 97.5% of the pikas’ diet with much smaller proportions being C_4_ (1.25%) and CAM (1.25%; Table S2).

### 3.2 Effect of talus habitat on plant diet richness

Model‐averaged estimates derived from the 90% model set agreed with the best approximating model with two variables: talus area cover and crevice depth, which were detected as significant predictors for diet richness; each having RI values of 1.0. Talus area has significant positive influence on plant diet richness. However, crevice depth showed a negative influence on plant richness (Table 2).

**Table 2:** Model‐averaged predictive models for effect of each parameter on plant diet richness with AIC < 2 and relative importance (RI) of each parameter

Parameter	Estimate (β) ± SE	Confidence interval		*Z* value	*p* value	RI
		Lower (2.5%)	Upper (97.5%)			
Intercept	1.65 ± 0.04	1.56	1.73	37.87	**0.001**	
Talus area cover	0.17 ± 0.04	0.07	0.27	3.50	**0.001**	1.00
Depth of crevices	−0.13 ± 0.04	−0.22	−0.03	2.78	**0.01**	1.00
Talus area cover X						
Depth of crevices	−0.009 ± 0.0 3	−0.03	0.16	0.27	0.78	0.29

The bold value indicates statistically significant *p* value which is less than 0.05.

## 4 DISCUSSION

This is the first study of the diet for any Himalayan pika species that uses noninvasive sampling and metabarcoding. It allowed us to quantify genus richness and revealed cryptic aspects of functional diversity and useful insights into niche partitioning across an elevational gradient. Our study provides an excellent example of how DNA metabarcoding can be used in understanding diet and feeding preferences of an elusive herbivore species found in fragmented alpine terrain and is applicable for other herbivores dependent on C_3_ and C_4_ plants. We found DNA metabarcoding outperformed traditional methods by revealing the huge diversity of plant genera consumed by pikas and their reliance on species endemic to the Himalayas. Earlier studies based on traditional visual and microscopic observation reported only 22 food plant genera for Royle's pika (Bhattacharyya et al., 2013), whereas our DNA metabarcoding results indicated more than 70 plant genera in the diet, which includes all of the 22 plant genera reported earlier. Forbs constituted the highest proportion of the diet. We demonstrate quantitative estimates of relative consumption of plant genera and capture fine‐scale distribution across an elevational gradient, which has been difficult to detect using traditional methods of diet analysis. In addition, diet plant richness in the premonsoon season varied significantly across sites and decreased in higher elevation sites. We also demonstrate the effects of habitat characteristics, such as talus area and depth of crevices between rocks, on the genus richness in the Royle's pika's diet.

### 4.1 Plant composition and seasonal variation in Royle's pika diet

Our study revealed that pikas exhibit dietary flexibility with high genus richness in their premonsoon diet at lower elevation, possibly due to a longer growing period in this habitat. Thick snow cover delays the beginning of the growing period of alpine plants in higher elevation areas (Inouye, 2008). Therefore, plants experience comparatively longer growing periods at lower elevations (Körner, 2003). However, the lack of precipitation and low temperature during the postmonsoon period leads to the end of the growing period for alpine plants. This may explain why no variation in diet plant richness with elevation was observed during the postmonsoon season. Compared to other Asian pika species such as Northern pika (*Ochotona hyperborean;* Khlebnikova, 1976; Revin & Boeskorov, 1990), Royle's pikas show a preference for plants such as forbs (e.g., *Potentila* spp., *Primula* spp. and *Anaphalis* spp.). The contribution of forbs in the pika's diet was found to be proportionately higher than its availability in the environment possibly due to the high nutrient value of forbs compared to other vegetation types, such as grasses, shrubs and trees (Bhattacharyya et al., 2013).

Talus‐dwelling *Ochotona* species usually exhibit prominent hay building activity in the summer to cache food for survival during the winter (Bhattacharyya & Ray, 2015; Hudson, Morrison, & Hik, 2008). This food caching helps in lowering the quantity of secondary metabolites during winter consumption (Dearing, 1997). However, Royle's pikas do not collect or store plants as haypiles, but instead feed on dry leaves of shrub and tree species which have low plant secondary metabolites only during the resource‐limited postmonsoon season. Himalaya experiences a relatively short snow covered period compared to other mountain regions, and food plants (mosses, lichens, dry leaves from trees and shrubs) are available during the postmonsoon and winter, which probably led to the weak hoarding behaviour observed in the Royle's pika. Its premonsoon diet is rich in nitrogen, moisture, low in secondary metabolites and probably more suitable for spring litter. Although, one of the caveats in this study was the inconsistent seasonal sampling across sites, which would have allowed us to explore seasonal differences within the sites more effectively.

### 4.2 Effect of habitat structure on plant diet richness

In western Himalayan Royle's pika populations, plant diet richness increased with large talus area, whereas depth of crevices between rocks had a negative effect on plant diet richness. Plant richness in pika diet increased with talus area as larger areas have higher plant diversity (area–richness relationship). Large talus areas provide good cover and habitat both in terms of food availability and refuge from predators. Large talus provides pikas with many escape routes from predators, allowing pikas to forage on surrounding meadows and reduce the dependence on the availability of local vegetation. This may explain the higher plant diet richness that was observed, as predation risk plays an important role in determining the food selection in Royle's pika (Bhattacharyya et al., 2013).

Predation risk has significant impacts on prey populations, either by direct predation‐mediated mortality or indirectly by altering their physiology and behaviour (Lima, 1998; Lima & Dill, 1990; Schmitz, Beckerman, & O'Brien, 1997; Sinclair & Arcese, 1995). Large talus areas with high rock cover and vegetation help in forage selection by reducing other constraints such as energy demands (Stephens & Krebs, 1986). Pikas balance predation risk associated with foraging activity against nutritional quality and availability of diet plants, a typical strategy found in central place foragers by exploiting nearby talus habitats (Bhattacharyya et al., 2013; Huntly et al., 1986; Morrison, Barton, Caputa, & Hik, 2004; Smith, Formozov, Hoffmann, Changlin, & Erbajeva, 1990). Large rock talus fields with crevices possibly act as escape cover from predators and allow pikas to access extended foraging grounds and diverse array of food plants, and hence have a positive influence on diet richness. Furthermore, Royle's pikas are sensitive to high temperature, and stable microclimate talus habitat serves as a refuge from harsh climate as well as predators (Bhattacharyya et al., 2014b). Royle's pika utilizes crevices in their talus habitat to build their nests (Bhattacharyya et al., 2015). The structure of rock talus and availability of small crevices (< 15 cm) appear to govern occupancy of Royle's pika habitat as it reduces predation risk; wide crevices probably make it easier for predators such as weasels and red foxes to catch pikas (Bhattacharyya et al., 2015). We found deep crevices had a negative effect on plant diet richness. The negative effect of deep crevices probably indicates foraging under fear response. The deep crevices often also have wider openings that increase predation risk from small size predators such as weasels and red foxes (Bhattacharyya et al., 2015). Hence, pikas inhabiting talus with deep crevices probably utilize the foraging ground less extensively and have a narrow choice of plants. This could be a mechanism behind the avoidance of such taluses as restricted diet can further reduce individual fitness. These findings are in line with previous studies on the Royle's pika and other talus‐dwelling pikas, which tend to forage close to the talus habitat where predation risk is the least, and venture out to open meadows to forage only when talus patches are well connected (Bhattacharyya et al., 2013; Holmes, 1991; Morrison et al., 2004; Roach, Huntly, & Inouye, 2001).

### 4.3 Ecological and evolutionary linkages in the Royle's pika diet

The majority (97.5%) of diet plants in this study were C_3_ food plants, followed by C_4_ (1.25%) and CAM (1.25%) plants. Previous research has shown that expansion of C_4_ plants with low nutrient availability might have led to the extinction and contraction of distributional range of herbivorous mammals which were dependent on C_3_ plants during late Miocene (Cerling, Ehleringer, & Harris, 1998; Ehleringer, Cerling, & Dearing, 2002; Ge et al., 2012; MacFadden & Ceding, 1994; Osborne, 2008; Osborne & Beerling, 2006). The prehistoric distribution of pikas was closely associated with distribution of their preferred C_3_ food plants, such as those with high protein and moisture content (Ge et al., 2012). Climate change has also been found to alter the distribution and abundance of alpine plants based on their temperature sensitivity (Scherrer & Körner, 2011). Replacement of C_3_ plants by C_4_ plants across large landscapes during the late Miocene resulted in the extinction of a high number of pika species (Ge et al., 2012). Gottfried et al. (2012) suggested that the thermophylization process could lead to the replacement of cold and moist environment plant species (e.g., C_3_ plants) with an abundance of warm and dry environment plant species, (e.g., C_4_ and CAM plants). Given that the Royle's pika diet consists of C_3_ plant species (e.g., *Potentila*,* Anaphalis*), the increase in temperature and rainfall patterns is bound to influence the distribution range of plant species as well as pikas. In the past 25 years (1982–2006), the Himalayan arc has experienced significant decreases in winter precipitation (17 mm), which has resulted in an increase in mean annual (0.04–0.08°C per year), spring (0.02–0.08°C per year) and winter temperatures (0.03–0.04°C per year; Shrestha, Gautam, & Bawa, 2012). This continued increase in temperature could have a significant effect on the distribution dynamics of C_3_ species and potentially influence plant–herbivore interactions at a microhabitat level.

Alpine species adapted to cold climatic conditions are more vulnerable to global warming (Hughes, 2000). Moritz et al. (2008) indicated significant changes in distributions and range contraction in mountain‐dwelling small mammals. Walther et al. (2002) predicted upslope range shifts in animals to cope with changing (warming) environmental conditions. However, habitat fragmentation might not allow small mammals such as pikas to move their ranges fast enough to track shifts in suitable microclimates (Ray et al., 2012; Schloss, Nuñez, & Lawler, 2012). Given that the Royle's pika can cope with physiological stress (e.g., hypoxia) in high‐elevation environments, the upper limit of the distribution of diet plants (> 4,500 m) could still pose a threat to their survival and fitness. Isolated high altitude mountain habitats, also known as “Sky‐islands,” differ significantly in environmental conditions from intervening valleys (Shepard & Burbrink, 2008). These intervening valleys were found to have significant impacts on the dispersal of cold‐adapted mountain‐dwelling species, such as pikas, across sky‐islands (Galbreath, Hafner, Zamudio, & Agnew, 2010). Hence, the Royle's pika would potentially need to travel across the lowland valleys to find favourable habitat to cope with the changing environment. Apart from high sensitivity to warm environments (Bhattacharyya et al., 2014b) and predation risk (Bhattacharyya et al., 2015), absence of diet plants below 1,500 m elevation might hamper such contemporary migration through lowland valleys. Furthermore, loss of distribution range and connectivity between habitat patches along with nutritional stress might also make small pika populations more vulnerable to both climatic changes as well as other threats, such as the susceptibility to infectious diseases (Biebach & Keller, 2009; Epstein, 2001; Hanski & Gilpin, 1991; Harvell et al., 2002).

India holds 28% of the flora which is endemic to the Himalayan region (Chitale, Behera, & Roy, 2014). We found a high dependency of the Royle's pika on endemic plants, and 53%–64% of dietary plant genera (e.g., *Geum* and *Fragaria*) are endemic to the Himalayan region. Increases in summer temperature and precipitation and the frequency of freeze–thaw cycles are predicted to have detrimental, multidimensional and spatially variable impact on these endemic alpine plants (Dolezal et al., 2016). Recent research has suggested around 23.9% to 41.34% reduction in the distribution of endemic plants in various biodiversity hotspots in Himalaya by 2080 (Chitale et al., 2014). Therefore, changes in the distribution and abundance of Himalayan endemic plants might have significant negative impacts on the overall nutritional ecology of the Royle's pika. Lack of a plant genetic reference library from the Himalayan region, especially from our study site, did not allow us to achieve species‐level identification of pika diet. Future research is needed to build a plant genetic resource reference library for insights to be gained at the plant species level.

## 5 CONCLUSIONS

This is the first study of the Royle's pika diet using DNA metabarcoding and successfully revealed feeding preferences in this climate‐sensitive herbivore. We were able to quantify the genus richness in the pika's diet in various habitats and at different elevations. We revealed the high dependency of the pika on C_3_ plants and plants endemic to the Himalayan region. We showed that seasonal differences in the diet are associated with elevation and that diet varies at different sites due to differences in topography and climate. A significant amount (> 89%) of the pika's diet consisted of forbs. The genus richness in the diet is strongly predicted by the size of the talus available to the pika for foraging, with large areas resulting in the highest levels of diversity observed in the diet. Crevice depth negatively influences plant diet richness due to increased predation risk. The continued increase in temperature could have a significant effect on the distribution of C_3_ plants and reduce the amount of plant species available on which the pika can feed, which would be likely to impact both pika numbers and their distribution, especially in lower elevation habitats.

## AUTHORS’ CONTRIBUTION

S.B. collected the samples and performed the laboratory work. D.A.D. and F.I. supervised the project. S.B., H.H. and F.I. analysed the data. S.B. and F.I. wrote the manuscript. D.A.D. and H.H. commented on the manuscript. All authors approved the final version of the manuscript.

## Data Availability

GPS locations of all sample collection points, R code and data for generalized linear modelling, DNA sequences generated during the study can be accessed in the Dryad database (https://doi.org/10.5061/dryad.nt40m53).
