# Supplementary material for: A diet rich in C3 plants reveals the sensitivity of an alpine mammal to climate change
Source: Mol Ecol. 2018 Sep 17;28(2):250–65. doi: 10.1111/mec.14842 (PMC6391869; doi:10.1111/mec.14842)
Supplement: Supplementary file 1 [file MEC-28-250-s001.pdf]

Supplementary information for:

A diet rich in C_3 plants reveals the sensitivity of an alpine mammal to climate change

Sabuj Bhattacharyya[1, 2], Deborah A. Dawson[2], Helen Hipperson[2], Farah Ishtiaq[1]

[1]Centre for Ecological Sciences, Indian Institute of Science, Bangalore, India
[2]Department of Animal and Plant Sciences, Western Bank, Sheffield, S10 2TN, UK

*Corresponding author: Sabuj Bhattacharyya, bhattacharyyasabuj@gmail.com

Table of contents

**Table S1:** Generalised Linear Models explaining factors affecting plant diet richness in Royle's pika following information-theoretic approach (AICc values=Akaike's information criterion, ΔAICc=the difference between the model indicated and the best model; the model with lowest AICc; K= number of fitted parameters; *Wi*=Akaike weights; TAT=total area of talus; NT= distance to nearest talus; ROT=rock cover; CDT= depth of crevices; FOT= forbs cover; GRT=grass cover; SHT=shrub cover; TRT=tree cover; TVCT=total vegetation cover; ASP=aspect; SLO=slope of plot; ELE=elevation)	**Page 2-4**
**Table S2**: List of plant genera detected through DNA metabarcoding using ITS2 and *rbcL* gene in faecal pellets of Royle's pika (PP= Photosynthetic Pathway; LD= Lower Distribution limit (in m); UD= Upper Distribution Limit (in m); EO= Evolutionary Origin; EOB= Elevation of observation (in m); PremC = Overall contribution in pre-monsoon diet; PostC= Overall contribution to post-monsoon diet; CAS: Centrasiatic; EHI: Himalayan Endemic; HOL: Holarctic; MAL: South East Asiatic Malaysian; SIJ: Sino Japanese or Eastern Asiatic; TRO: Tropical; YUN: South East Chinese; HAK= Har ki doon; MAD= Madmaheshwar; TUN=Tungnath; RUD=Rudranath; NAN=Bedni-roopkund; Prm= Pre-monsoon, Pom=Post-monsoon).	**Page 5-28**
**Figure S1**: Plant diet richness detected in pre-monsoon (Premon) and post-monsoon (Postmon) season in Royle's pika (HAK= Har ki doon; MAD= Madmaheshwar; TUN=Tungnath; RUD=Rudranath; NAN=Bedni-roopkund). We found pre-monsoon plant composition in diet varied significantly (delta obs=2.54, delta exp. = 2.56, A=0.006, $P<0.05$)	**Page 29**
**Figure S2**: Contribution of vegetation groupsin faecal sample (Faecal) and environment (Environ) of survey plot (HAK= Har ki doon; MAD= Madmaheshwar; TUN=Tungnath; RUD=Rudranath; NAN=Bedni-roopkund; FO=forb, GR=grass, SH=shrub, TR=tree)	**Page 30**
**References**	**Page 31-33**

Table S1: Generalised Linear Models, factors affecting species richness in Royle's pika following information-theoretic approach

(AICc values=Akaike's information criterion,
ΔAICc=the difference between the model indicated and the best model; the model with lowest AICc;
K= number of fitted parameters;
Wi=Akaike weights;
TAT=total area of talus;
NT= distance to nearest talus;
ROT=rock cover;
CDT= depth of crevices;
FOT= forbs cover;
GRT=grass cover;
SHT=shrub cover;
TRT=tree cover;
TVCT=total vegetation cover;
ASP=aspect;
SLO=slope of plot;
ELE=elevation)

Model no	Ecological Hypothesis Tested	Model structure (predictor)	df	Log lik	AICc	ΔAICc	Wi
1	Predation risk	TAT+CDT	3	-303.0	612.1	0	0.478
2	Predation risk	TAT*CDT	4	-302.8	613.9	1.81	0.193
3	Predation risk	TAT*CDT+NTT	5	-302.0	614.5	2.37	0.146
4	Predation risk	TAT*CDT+NTT+ROT	6	-301.8	616.3	4.2	0.059
5	Food availability, predation risk	TAT*CDT+NTT+ROT+FOT	7	-301.2	617.3	5.2	0.035
6	Predation risk	TAT	2	-307.0	618.1	5.96	0.024
7	Food availability, habitat topography, predation risk	TAT*CDT+NTT+ROT+FOT+SHT+TRT+GRT+ELET	11	-296.9	618.3	6.16	0.022
8	Food availability, predation risk	TAT*CDT+NTT+ROT+FOT+SH	8	-300.6	618.5	6.43	0.019

Model no	Ecological Hypothesis Tested	Model structure (predictor)	df	Log lik	AICc	ΔAICc	Wi
		T					
9	Food availability, predation risk	TAT*CDT+NTT+ROT+FOT+SHT+GRT	9	-300.4	620.5	8.35	0.007
10	Habitat topography	ELE	2	-308.6	621.3	9.23	0.005
11	Food availability	TRT	2	-308.7	621.5	9.4	0.004
12	Predation risk	SHT	2	-310.1	624.2	12.1	0.001
13	Predation risk	NTT	2	-310.3	624.7	12.58	0.001
14	No impact	Null model	1	-311.4	624.9	12.78	0.001
15	Food availability, Habitat topography, predation risk	TAT+NTT+ROT+SLO*FOT+SLO*SHT+ SLO*TRT+SLO*GRT+ELET+ASP	20	-288.3	625	12.89	0.001
16	Predation risk	CDT	2	-310.7	625.6	13.47	0.001
17	Habitat topography	SLO	2	-310.8	625.7	13.58	0.001
18	Food availability	GRT	2	-311.2	626.4	14.32	0
19	Food availability	FOT	2	-311.3	626.7	14.6	0
20	Predation risk	ROT	2	-311.4	627	14.84	0
21	Food availability, habitat topography, predation risk	TAT*CDT+NTT+ROT+FOT+SHT+TRT+GRT +ELET+ASP+SLO	16	-295.3	627.9	15.78	0
22	Food availability, habitat topography, predation risk	TAT*CDT+NTT+ROT+FOT+SHT+TRT+GRT +ELET+ASP	17	-295.5	631	18.9	0
23	Food availability, habitat topography, predation risk	NTT+ROT+FOT+SHT+GRT+TRT+ELET+ASP+SLO	15	-298.4	631.5	19.36	0
24	Food availability, habitat topography, predation risk	TAT*FOT+TAT*SHT+ TAT*TRT+TAT*GRT+NTT+ROT+ELET+ASP+SLO	20	-291.5	631.5	19.37	0

Model no	Ecological Hypothesis Tested	Model structure (predictor)	df	Log lik	AICc	ΔAICc	Wi
25	Food availability, habitat topography, predation risk	TAT+NTT+ROT+ASP*FOT+ASP*SHT+ASP*TRT+ ASP*GRT+ELET+SLO	28	-278.8	631.5	19.42	0
26	Habitat topography	ELET+ASP+SLO	9	-306.4	632.4	20.32	0
27	Habitat topography	ASP	7	-309.0	633	20.91	0
28	Food availability, habitat topography	SHT+GRT+ELET+ASP+SLO	11	-304.5	633.4	21.33	0
29	Food availability, habitat topography	GRT+ELET+ASP+SLO	10	-306.1	634.3	22.2	0
30	Habitat topography	ASP+SLO	8	-308.6	634.5	22.38	0
31	Food availability, habitat topography	FOT+SHT+GRT+ELET+ASP+SLO	12	-304.5	635.9	23.79	0
32	Food availability, habitat topography, predation risk	ROT+FOT+SHT+GRT+ELET+ASP+SLO	13	-304.5	638.4	26.3	0

Table S2: List of the plant genera detected using DNA metabarcoding of the ITS2 and *rbcL* regions amplified from DNA extracted from the faecal pellets of the Royle's pika

PP= Photosynthetic Pathway; LD= Lower Distribution limit (in m); UD= Upper Distribution Limit (in m); EO= Evolutionary Origin; EOB= Elevation of observation (in m); PremC = Overall contribution in pre-monsoon diet; PostC= Overall contribution to post-monsoon diet; CAS: Centrasiatic; EHI: Himalayan Endemic; HOL: Holarctic; MAL: South East Asiatic Malaysian; SIJ: Sino Japanese or Eastern Asiatic; TRO: Tropical; YUN: South East Chinese; HAK= Har ki doon; MAD= Madmaheshwar; TUN=Tungnath; RUD=Rudranath; NAN=Bedni-roopkund; Prm= Pre-monsoon, Pom=Post-monsoon)

Site	Season	Family	Genus	UD	LD	EOB	EO	PP	PrmC	PomC	Reported in other Pika species	References
RUD	Pom		Angelica	2500	1500	3491	EHI			25	*O. princeps*	Elliott, 1980
TUN	Prm		Bupleurum	4000	2000	3228	EHI		3.7		*O. alpina*	Sun et al. 2008
NAN	Prm		Pimpinella	3500	2000	3269	SIJ		6.25			Sage 2016
NAN	Prm	Apiaceae				3243		C3				
TUN	Pom		Selinum			3380	EHI			7.69		Sage 2016
TUN	Pom			4500	3000	3455						
TUN	Pom		Tordyliopsis			3455	EHI			3.85		
TUN	Prm				2600	2712						Liu et al., 2008, 2009a, c;
TUN	Prm	Asteraceae	Anaphalis	5000		3326		C3	55.55		*O. cansus*	Su et al., 2004
TUN	Prm					3269						
TUN	Prm					3269						
TUN	Prm					3269						

Site	Season	Family	Genus	UD	LD	EOB	EO	PP	PrmC	PomC	Reported in other Pika species	References
TUN	Prm					2771						
TUN	Prm					3228						
TUN	Prm					2712						
TUN	Prm					3444						
TUN	Prm					2861						
TUN	Prm					3314						
TUN	Prm					2861						
TUN	Prm					3486						
TUN	Prm					2712						
TUN	Prm					2712						
MAD	Prm					3897						
MAD	Prm					3913						
MAD	Prm					3893						
MAD	Prm					3695						
MAD	Prm					3695			76.92			
MAD	Prm					3549						
MAD	Prm					3552						
MAD	Prm					3608						
MAD	Prm					3568						
MAD	Prm					3554						
NAN	Prm					3223						
NAN	Prm					3223			15.62			
NAN	Prm					3244						
NAN	Prm					3269						

Site	Season	Family	Genus	UD	LD	EOB	EO	PP	PrmC	PomC	Reported in other Pika species	References
NAN	Prm					3243						
TUN	Pom					3214						
TUN	Pom					3196						
TUN	Pom					3149						
TUN	Pom					3455						
TUN	Pom					3455						
TUN	Pom					3380						
TUN	Pom					3380						
TUN	Pom					3021				61.54		
TUN	Pom					2933						
TUN	Pom					2841						
TUN	Pom					2704						
TUN	Pom					2841						
TUN	Pom					3256						
TUN	Pom					2911						
TUN	Pom					2847						
TUN	Pom					2847						
HAK	Pom					2623				12.5		
RUD	Pom					3110						
RUD	Pom					3491				100		
RUD	Pom					3156						
RUD	Pom					3496						
NAN	Prm		Aster	5000	3000	3223		C3	3.12		*O. dauurica,O. curzoniae,*	Bannikov, 1954

Site	Season	Family	Genus	UD	LD	EOB	EO	PP	PrmC	PomC	Reported in other Pika species	References
											O. rufescens	,Liu et al., 2009 c,Lay, 1967
TUN	Pom		Cirsium	4000	1000	3455	EHI	C3		3.85	*O. princeps*	Johnson, 1967; Elliott, 1980
NAN	Prm		Melanoseris			3269		C3	3.12			Sage 2016
TUN	Prm		Myriactis	4000	2000	2645	CAS	C3	3.7			Sage 2016
TUN	Prm					2861			7.4		*O. curzoniae*	Ye, 2006; Jiang and Xia, 1985
TUN	Prm					2712						
MAD	Prm					3549			23.07			
MAD	Prm					3545						
MAD	Prm					3608						
MAD	Prm		Taraxacum	5000	3000	3561	YUN	C3				
TUN	Pom					3149				19.23		
TUN	Pom					3090						
TUN	Pom					3455						
TUN	Pom					3455						
TUN	Pom					3256						
MAD	Prm	Balsaminaceae	Impatiens	3500	3000	3545	EHI	C3	7.69		*O. alpina*	Sun et al. 2008
NAN	Prm					3223			6.25		*O. hyperborea*	Bannikov, 1954
NAN	Prm	Berberidaceae	Berberis	4000	2000	3243	EHI	C3				
RUD	Pom					3491				25		
TUN	Prm	Betulaceae	Alnus	3600	500	2771	MAL	C3	3.7			Sage 2016
NAN	Prm	Boraginaceae	Hackelia	4000	2500	3269	EHI	C3	3.12			Sage 2016
MAD	Prm	Boraginaceae	Myosotis	3500	2000	3685	HOL	C3	7.69			Sage 2016

Site	Season	Family	Genus	UD	LD	EOB	EO	PP	PrmC	PomC	Reported in other Pika species	References
NAN	Prm	Brassicaceae	Cardamine	5000	2000	3269	HOL	C3	3.12			Sage 2016
MAD	Prm	Campanulaceae	Cyananthus	5000	3000	3608	EHI	C3	7.69			Sage 2016
TUN	Pom					3455				3.85		
NAN	Prm	Caprifoliaceae	Lonicera	4500	2000	3269	YUN	C3	3.12		*O. alpina*<br>*O. princeps*<br>*O. hyperborea*	Liu et al., 2009c<br>Sun et al. 2008<br>Bannikov, 1954;<br>Millar and Zwickel, 1972<br>Gliwicz et al., 2006
TUN	Prm					3444						
TUN	Prm					3269			11.11			
TUN	Prm	Caprifoliaceae	Viburnum	3000	1500	2861	YUN	C3				Sage 2016
TUN	Pom					2911				3.85		
RUD	Pom					3156				25		
TUN	Prm					2645			3.7			
MAD	Prm					3685						
MAD	Prm		Cerastium	5000	3000	3557	HOL	C3	15.38			Sage 2016
NAN	Prm	Caryophyllaceae				3223						
NAN	Prm					3223			9.37			
NAN	Prm					3243						
TUN	Prm		Gypsophila	5000	2000	2861	EHI	C3	3.7			Sage 2016

Site	Season	Family	Genus	UD	LD	EOB	EO	PP	PrmC	PomC	Reported in other Pika species	References
NAN	Prm		Sagina	4000	2000	3243	HOL	C3	3.12			Sage 2016
TUN	Prm					2645			7.4			
TUN	Prm					2861						
NAN	Prm		Stellaria	3000	2000	3223	YUN	C3	6.25			Sage 2016
NAN	Prm					3243						
TUN	Pom					2847				3.85		
NAN	Prm	Crassulaceae	Crassula			3269		CAM	3.12			Gravatt et al 1992
NAN	Prm	Cruciferae	Brassica	4000	2500	3269		C3				Sage 2016
TUN	Prm					3314					*O. princeps,O. dauurica,*	Elliott, 1980; Rausch, 1962;Ognev, 1940
TUN	Prm					2861			11.11		*O. curzoniae,O. collaris,O. pallasi*	Sun et al. 2008; Bannikov, 1954:Borisova et al. 2001;Jiang and Xia; 1985
		Cyperaceae	Carex	5000	2000		EHI	C3				
TUN	Prm					3269						
MAD	Prm					3695						
MAD	Prm					3549			23.07			
MAD	Prm					3552						
NAN	Prm					3223						
NAN	Prm					3283			9.37			
NAN	Prm					3243						

Site	Season	Family	Genus	UD	LD	EOB	EO	PP	PrmC	PomC	Reported in other Pika species	References
TUN	Pom					3149						
TUN	Pom					2704				15.38		
TUN	Pom					3256						
TUN	Pom					2847						
RUD	Pom					3491				50		
RUD	Pom					3496						
MAD	Prm		Cassiope	5000	3000	3531	EHI	C3	7.69		*O. collaris*	Rausch, 1962
TUN	Prm					2712						
TUN	Prm					2771			11.11			
TUN	Prm					2861						
TUN	Pom		Gaultheria	4000	2000	3380	EHI	C3			*O. princeps*	Elliott, 1980
TUN	Pom					3455						
TUN	Pom					2933				19.23		
TUN	Pom					2704						
TUN	Pom	Ericaceae				2841						
TUN	Prm		Lyonia	3000	1000	2771	MAL	C3	3.7			Sage 2016
RUD	Pom					3455				25		
TUN	Prm					3269	IND, SIJ		3.7			
TUN	Pom		Rhododendron	4000	1500	3214	IND, SIJ	C3			*O. collaris*	Rausch, 1962
TUN	Pom					3380	IND, SIJ			11.5		
TUN	Pom					3021	IND, SIJ					
TUN	Prm	Fagaceae	Quercus	3500	2000	2771	YUN	C3	3.7		*O. alpina*	Sun et al. 2008

Site	Season	Family	Genus	UD	LD	EOB	EO	PP	PrmC	PomC	Reported in other Pika species	References
TUN	Prm	Gentianaceae	Gentiana	5000	2500	3269		C3	3.7		*O. curzoniae*	Fan et al., 1995
						3269					*O. dauurica*	
TUN	Pom	Geraniaceae	Geranium	3000	2000	3269		C3		3.85	*O. alpina*	Bannikov, 1954
TUN	Prm					3269	SIJ		3.7			
TUN	Pom	Hypericaceae	Hypericum	3000	500	3455	MAL	C3		3.85	*O. alpina*	Sun et al. 2008
TUN	Pom	Lamiaceae	Prunella	3500	2000	3455	HOL					
TUN	Pom					3214						
TUN	Pom					3149						
TUN	Pom		Circaea	5000	3000	3455	HOL	C3		23.08		Sage 2016
TUN	Pom					2911						
TUN	Pom					2704						
TUN	Pom					2911						
TUN	Prm					3326						
TUN	Prm					3269						
TUN	Prm	Onagraceae				3228			18.51			
TUN	Prm					2712						
TUN	Prm					3486						
MAD	Prm		Epilobium	4000	3000	3531	YUN	C3			*O. collaris*	Rausch, 1962
MAD	Prm					3695			23.07			
MAD	Prm					3554						
NAN	Prm					3223			6.25			
NAN	Prm					3223						
TUN	Pom					3214				7.69		
TUN	Pom					2847						

Site	Season	Family	Genus	UD	LD	EOB	EO	PP	PrmC	PomC	Reported in other Pika species	References
TUN	Prm		Prochetus			2704		C3	3.7			Dearing, 1997;
TUN	Prm	Papilionaecae	Trifolium	3000	1500	2645	HOL		7.4		*O.princeps*	Johnson, 1967
TUN	Prm					2861						
MAD	Prm					3557						
MAD	Prm					3552						
MAD	Prm					3545			38.46			
MAD	Prm					3608						
MAD	Prm					3590						
MAD	Prm		Agrostis	5000	2000	3561	EHI	C3			*O. alpina*	Sun et al. 2008
TUN	Prm					3228			7.4			
TUN	Prm					2861						
TUN	Pom					3214						
TUN	Pom	Poaceae				3149				15.38		
TUN	Pom					3455						
TUN	Pom					3256						
MAD	Prm					3549			7.69			
NAN	Prm		Alopecurus	3000	2000	3223	HOL	C3	3.13			Osborne et al 2014
TUN	Pom					3380				3.85		
TUN	Prm		Calamagrostis	5000	3000	2771	EHI		3.7		*O. alpina,* *O. princeps*	Bannikov, 1954,Elliott, 1980
TUN	Prm					3314						
TUN	Prm		Danthonia	4500	2000	3269	EHI	C3	18.51			Osborne et al 2014
TUN	Prm					3228						

Site	Season	Family	Genus	UD	LD	EOB	EO	PP	PrmC	PomC	Reported in other Pika species	References
TUN	Prm					3444						
TUN	Prm					2712						
MAD	Prm					3552						
MAD	Prm					3554			15.38			
MAD	Prm					3608						
MAD	Prm					3561						
TUN	Pom					3214						
TUN	Pom					3149				15.38		
TUN	Pom					3455						
TUN	Pom					3256						
HAK	Pom					3443				12.5		
MAD	Prm		Deschampsia	5000	3000	3685	HOL		7.69		*O. princeps*	Johnson, 1967, Dearing 1995
MAD	Prm		Festuca	3000	2000	3552	HOL	C3	7.69		*O. curzoniae* *O. hyperborea*	Jiang and Xia, 1985, Bannikov 1954
TUN	Pom		Helictotrichon	3000	2000	3455	YUN	C3		3.85		Osborne et al 2014
TUN	Prm					2645			7.4			
TUN	Prm		Muhlenbergia			2771		C4	7.4			Osborne et al 2014
TUN	Pom					2704				3.85		
TUN	Prm					2645					*O. curzoniae*	Fan et al., 1995 ;
TUN	Prm		Poa	4000	2000	2712	HOL	C3	55.55		*O. curzoniae*	Liu et al., 2009a
TUN	Prm					3326					*O. dauurica*	

Site	Season	Family	Genus	UD	LD	EOB	EO	PP	PrmC	PomC	Reported in other Pika species	References
TUN	Prm					3314					*O. princeps*	
TUN	Prm					3269					*O. curzoniae*	
TUN	Prm					3269						
TUN	Prm					3269						
TUN	Prm					2771						
TUN	Prm					3228						
TUN	Prm					3228						
TUN	Prm					2712						
TUN	Prm					3326						
TUN	Prm					3444						
TUN	Prm					2861						
TUN	Prm					3314						
TUN	Prm					3269						
TUN	Prm					3269						
TUN	Prm					3269						
TUN	Prm					2861						
TUN	Prm					3486						
TUN	Prm					2712						
MAD	Prm					3897						
MAD	Prm					3913			23.07			
MAD	Prm					3893						
NAN	Prm					3223						
NAN	Prm					3223			25			
NAN	Prm					3244						

Site	Season	Family	Genus	UD	LD	EOB	EO	PP	PrmC	PomC	Reported in other Pika species	References
NAN	Prm					3269						
NAN	Prm					3269						
NAN	Prm					3243						
NAN	Prm					3243						
NAN	Prm					3243						
TUN	Pom					3196						
TUN	Pom					3149						
TUN	Pom					3130						
TUN	Pom					3090						
TUN	Pom					3380						
TUN	Pom					3380						
TUN	Pom					3021				57.69		
TUN	Pom					2933						
TUN	Pom					2841						
TUN	Pom					2704						
TUN	Pom					2841						
TUN	Pom					3256						
TUN	Pom					2911						
TUN	Pom					2911						
HAK	Pom					2623				12.5		
RUD	Pom					3156						
RUD	Pom					3110				100		
RUD	Pom					3491						
RUD	Pom					3496						

Site	Season	Family	Genus	UD	LD	EOB	EO	PP	PrmC	PomC	Reported in other Pika species	References
TUN	Prm		Trisetum	3500	3000	2771	EHI		3.7		*O. dauurica*<br>*O. princeps*	Bannikov, 1954<br>Johnson, 1967
MAD	Prm		Bistorta	4500	2500	3531		C3	7.69		*O. princeps*	Dearing, 1996, 1997
MAD	Prm					3561						
NAN	Prm		Koenigia			3223		C3	3.12			Sage 2016
TUN	Pom					3455				3.85		
MAD	Prm		Rheum	4500	3000	3531	EHI	C3	7.69			Sage 2016
MAD	Prm	Polygonaceae	Rhodiola	4000	1000	3531	EHI	C3				Sage 2016
NAN	Prm		Rumex	4000	1000	3223	HOL	C3	3.12			Sage 2016
TUN	Pom					3149						
TUN	Pom		Persicaria			3455		C3		15.38		Sage 2016
TUN	Pom					2704						
TUN	Pom					2847						
NAN	Prm					3223						
NAN	Prm		Lysimachia	3000	2500	3269	YUN		12.5			
NAN	Prm					3243						
NAN	Prm					3243						
TUN	Prm			5000		2645						
TUN	Prm	Primulaceae				3326						
TUN	Prm		Primula		1500	3269	EHI		51.85			
TUN	Prm					3269						
TUN	Prm					2771						
TUN	Prm					3228						

Site	Season	Family	Genus	UD	LD	EOB	EO	PP	PrmC	PomC	Reported in other Pika species	References
TUN	Prm					2712						
TUN	Prm					3326						
TUN	Prm					3444						
TUN	Prm					3314						
TUN	Prm					3269						
TUN	Prm					2861						
TUN	Prm					3486						
TUN	Prm					2712						
MAD	Prm					3897						
MAD	Prm					3314						
MAD	Prm					3913						
MAD	Prm					3893						
MAD	Prm					3695						
MAD	Prm					3695						
MAD	Prm					3685			100			
MAD	Prm					3549						
MAD	Prm					3549						
MAD	Prm					3557						
MAD	Prm					3608						
MAD	Prm					3590						
MAD	Prm					3588						
NAN	Prm					3223						
NAN	Prm					3223			21.87			
NAN	Prm					3244						

Site	Season	Family	Genus	UD	LD	EOB	EO	PP	PrmC	PomC	Reported in other Pika species	References
NAN	Prm					3269						
NAN	Prm					3243						
NAN	Prm					3243						
NAN	Prm					3243						
TUN	Pom					3196						
TUN	Pom					3149						
TUN	Pom					3090						
TUN	Pom					3380						
TUN	Pom					3380						
TUN	Pom					3021				46.15		
TUN	Pom					2933						
TUN	Pom					2841						
TUN	Pom					2841						
TUN	Pom					2911						
TUN	Pom					2847						
TUN	Pom					2847						
HAK	Pom					2623				12.5		
RUD	Pom					3156						
RUD	Pom					3110				100		
RUD	Pom					3496						
RUD	Pom					3491						
NAN	Prm					3223			3.12		*O. cansus*	
TUN	Pom	Ranunculaceae	Anemone	4500	2000	3455	EHI	C3		7.69	*O. curzoniae*	Su et al., 2004 ; Jiang and Xia, 1985
TUN	Pom					3256						

Site	Season	Family	Genus	UD	LD	EOB	EO	PP	PrmC	PomC	Reported in other Pika species	References
NAN	Prm		Caltha	4500	2000	3223	EHI	C3	3.12		*O. hyperborea*	Gliwicz et al., 2006
TUN	Prm					3269						
TUN	Prm					3228						
TUN	Prm					3228			18.51			
TUN	Prm					2712						
TUN	Prm		Oxygraphis	6000	2000	2861	EHI	C3				Sage 2016
MAD	Prm					3531						
MAD	Prm					3685			30.76			
MAD	Prm					3557						
TUN	Pom					3214				3.85		
TUN	Prm					3269						
TUN	Prm					3269						
TUN	Prm					3269						
TUN	Prm					3228						
TUN	Prm					3228						
TUN	Prm					3228			40.74			
TUN	Prm					2712						
TUN	Prm		Ranunculus	6000	2500	3444	SIJ	C3			*O. cansus*	Su et al., 2004
TUN	Prm					2861						
TUN	Prm					3269						
TUN	Prm					2712						
MAD	Prm					3695						
MAD	Prm					3685			61.53			
MAD	Prm					3549						

Site	Season	Family	Genus	UD	LD	EOB	EO	PP	PrmC	PomC	Reported in other Pika species	References
MAD	Prm					3554						
MAD	Prm					3545						
MAD	Prm					3608						
MAD	Prm					3385						
MAD	Prm					3554						
NAN	Prm					3243			3.12			
TUN	Pom					3214						
TUN	Pom					3256						
TUN	Pom					2911				19.23		
TUN	Pom					2847						
TUN	Pom					2847						
HAK	Pom					3453				12.5		
RUD	Pom					3110				25		
TUN	Prm					3269						
TUN	Prm					2771						
TUN	Prm					2861			18.51			
TUN	Prm					3486						
TUN	Prm					2712						
NAN	Prm	Rosaceae	Fragaria	5000	2000	3269	EHI	C3			*O. princeps*	Elliott, 1980
NAN	Prm					3243			9.37			
NAN	Prm					3243						
TUN	Pom					3214						
TUN	Pom					3149				26.92		
TUN	Pom					3090						

Site	Season	Family	Genus	UD	LD	EOB	EO	PP	PrmC	PomC	Reported in other Pika species	References
TUN	Pom					3455						
TUN	Pom					2841						
TUN	Pom					3256						
TUN	Pom					2911						
RUD	Pom					3496				25		
TUN	Prm					2712			3.7			
MAD	Prm		Geum	3500	2500	3897	EHI	C3	15.38		*O. princeps*	Johnson, 1967
MAD	Prm					3531			15.38			
TUN	Pom					3256				3.85		
TUN	Prm					2712					*O. curzoniae*	
TUN	Prm					2712					*O. cansus*	
TUN	Prm					3326					*O. dauurica*	
TUN	Prm					3314					*O. princeps*	
TUN	Prm					3314					*O. pallasi*	
TUN	Prm					3269						
TUN	Prm					3269						
TUN	Prm		Potentila	5000	1500	3269	EHI	C3	40.74			Liu et al., 2008, 2009b; Jiang and Xia, 1985 ; Borisova et al. 2001;Su et al., 2004;Bannikov, 1954;Johnson, 1967 ;Elliott, 1980 ; Su et al., 2004 ; Millar and Zwickel, 1972;Liu et al., 2009c;Wang et al., 1992
TUN	Prm					3228						
TUN	Prm					3228						
TUN	Prm					3228						
TUN	Prm					2712						
TUN	Prm					3326						
TUN	Prm					3444						
TUN	Prm					2861						

Site	Season	Family	Genus	UD	LD	EOB	EO	PP	PrmC	PomC	Reported in other Pika species	References
TUN	Prm					3269						
TUN	Prm					2861						
TUN	Prm					3486						
TUN	Prm					2712						
TUN	Prm					2712						
MAD	Prm					3897						
MAD	Prm					3913						
MAD	Prm					3531						
MAD	Prm					3531						
MAD	Prm					3695						
MAD	Prm					3695						
MAD	Prm					3685						
NAN	Prm					3223						
NAN	Prm					3223						
NAN	Prm					3223						
NAN	Prm					3223						
NAN	Prm					3223						
NAN	Prm					3269			34.37			
NAN	Prm					3269						
NAN	Prm					3283						
NAN	Prm					3243						
NAN	Prm					3243						
NAN	Prm					3243						
TUN	Pom					3214				34.62		

Site	Season	Family	Genus	UD	LD	EOB	EO	PP	PrmC	PomC	Reported in other Pika species	References
TUN	Pom					3149						
TUN	Pom					3149						
TUN	Pom					3130						
TUN	Pom					3130						
TUN	Pom					3090						
TUN	Pom					3455						
TUN	Pom					3380						
TUN	Pom					3021						
HAK	Pom					2623				12.5		
RUD	Pom					3156						
RUD	Pom					3110				100		
RUD	Pom					3110						
RUD	Pom					3496						
TUN	Prm		Rosa	4000	2000	2771	EHI	C3	3.7		*O. alpina*	Ognev, 1940
NAN	Prm					3269			3.12			
TUN	Prm					2645					*O. alpina*	Sun et al. 2008
TUN	Prm					3269					*O. hyperborea*	Gliwicz et al., 2006
TUN	Prm		Rubus	4000	2000	3228	EHI	C3	22.22		*O. princeps*	Johnson, 1967
TUN	Prm					2712						
TUN	Prm					3486						
TUN	Prm					2712						
MAD	Prm					3695			23.07			

Site	Season	Family	Genus	UD	LD	EOB	EO	PP	PrmC	PomC	Reported in other Pika species	References
MAD	Prm					3549						
NAN	Prm					3243			3.12			
TUN	Pom					3380						
TUN	Pom					2704				11.54		
TUN	Pom					2911						
HAK	Pom					2623				12.5		
RUD	Pom					3496				25		
TUN	Prm					3269			7.4			
TUN	Prm					3228						
MAD	Prm					3531			7.69			
NAN	Prm					3223			3.12			
TUN	Pom		Sibbaldia	4500	3000	3149	HOL	C3				Sage 2016
TUN	Pom					3130				15.38		
TUN	Pom					3090						
TUN	Pom					3455						
TUN	Prm					2861			3.7		*O. princeps*	Elliott, 1980
NAN	Prm	Rubiaceae	Galium	3000	2000	3223	EHI	C3	6.25		*O. hyperborea*	Bannikov, 1954 ; Liu et al., 2009c
NAN	Prm					3269					*O. curzoniae*	
TUN	Pom	Salicaceae	Salix	3000	2500	3380		C3		3.85	*O. dauurica*	Bannikov, 1954

Site	Season	Family	Genus	UD	LD	EOB	EO	PP	PrmC	PomC	Reported in other Pika species	References
											O. princeps	Johnson, 1967 ; Rausch, 1962; Millar and Zwickel, 1972
RUD	Pom					3156					*O. collaris*	
TUN	Prm					2771			7.4			
TUN	Prm					2712						
NAN	Prm		Bergenia	4000	2000	3223	EHI		3.12			
TUN	Pom	Saxifragaceae				3380		C3		7.69		
TUN	Pom					3455						
MAD	Prm		Saxifraga	5000	3000	3531	EHI		7.69		*O. alpina*	Ognev, 1940
TUN	Pom					3455				3.85		
TUN	Prm					3269						
TUN	Prm					3269						
TUN	Prm					2712			22.22			
TUN	Prm					2861						
TUN	Prm					3486						
TUN	Prm	Scrophulariaceae	Hemiphragma	3000	2000	2712	MAL	C3				Sage 2016
NAN	Prm					3243						
NAN	Prm					3243			9.37			
NAN	Prm					3243						
TUN	Pom					3380				7.69		
TUN	Pom					2841						

Site	Season	Family	Genus	UD	LD	EOB	EO	PP	PrmC	PomC	Reported in other Pika species	References
TUN	Prm					3228			3.7			
MAD	Prm					3685						
MAD	Prm					3557			30.76			
MAD	Prm					3545						
MAD	Prm		Veronica	2500	2000	3590	YUN	C3				Sage 2016
TUN	Pom					3214						
TUN	Pom					3149				15.38		
TUN	Pom					3455						
TUN	Pom					3256						
TUN	Prm					3269						
TUN	Prm					3269						
TUN	Prm					2712			18.51			
TUN	Prm					2861						
TUN	Prm	Orobanchaceae	Pedicularis	4000	3000	3486	EHI	C3			*O. dauurica*	Bannikov, 1954
MAD	Prm					3552			15.38			
MAD	Prm					3552						
NAN	Prm					3243			3.12			
TUN	Pom					2841				3.85		
TUN	Pom	Urticaceae	Pilea	4000	2000	3149	YUN	C3				Sage 2016
NAN	Prm		Urtica	6000	2000	3269	HOL	C3	3.12		*O. princeps*	Johnson, 1967
NAN	Prm	Violaceae	Viola	4000	2000	3243	HOL	C3	3.12			Sage 2016

Number of unamplified sample	TUN -Prm	15
	TUN-Pom	14
	NAN	11
	MAD	6
	HAK	10
	RUD	4

Figure S1: Plant diet richness detected in pre-monsoon (premon) and post-monsoon (postmon) season in Royle's pika (HAK= Har ki doon; MAD= Madmaheshwar; TUN=Tungnath; RUD=Rudranath; NAN=Bedni-roopkund). We found pre-monsoon plant composition in diet varied significantly (delta obs=2.54, delta exp. = 2.56, A=0.006, *P*<0.05)

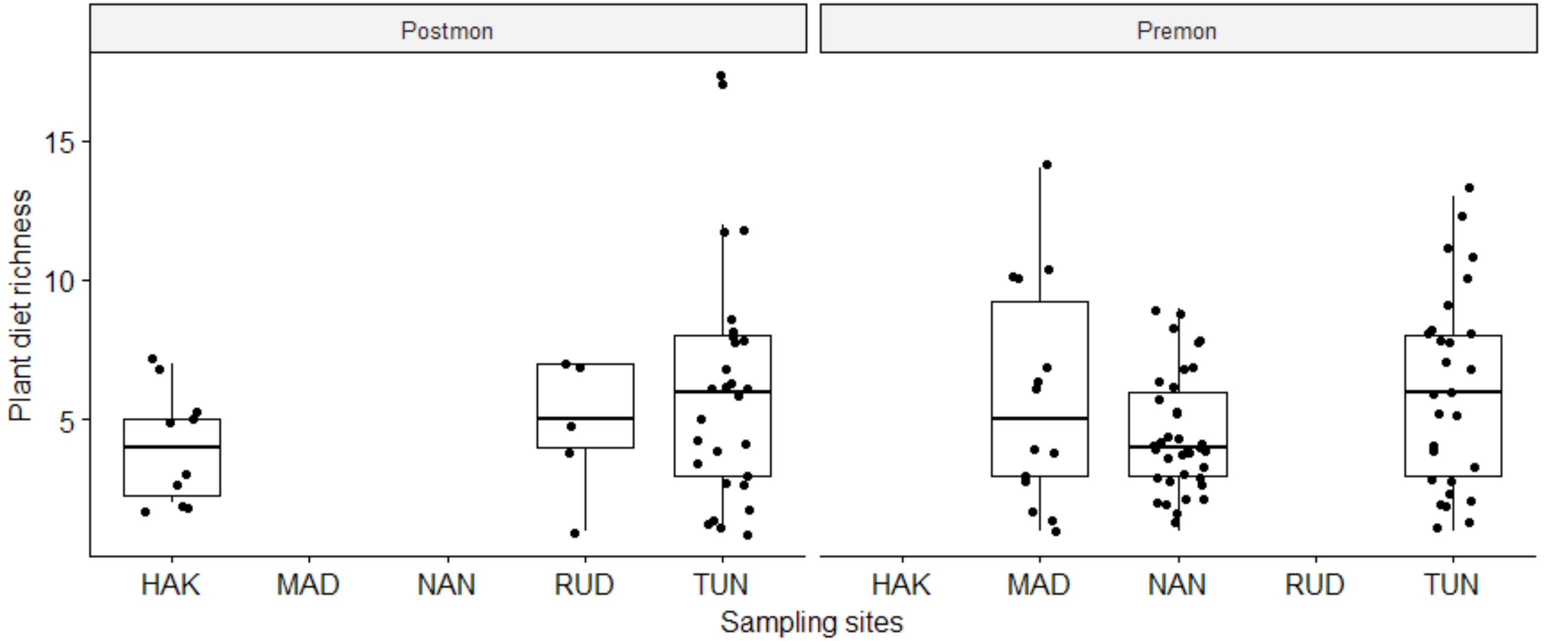

Figure S2: Contribution of vegetation groups in faecal sample (Faecal) and environment (Environ) of survey plot (HAK= Har ki doon; MAD= Madmaheshwar; TUN=Tungnath; RUD=Rudranath; NAN=Bedni-roopkund; FO=forb, GR=grass, SH=shrub, TR=tree)

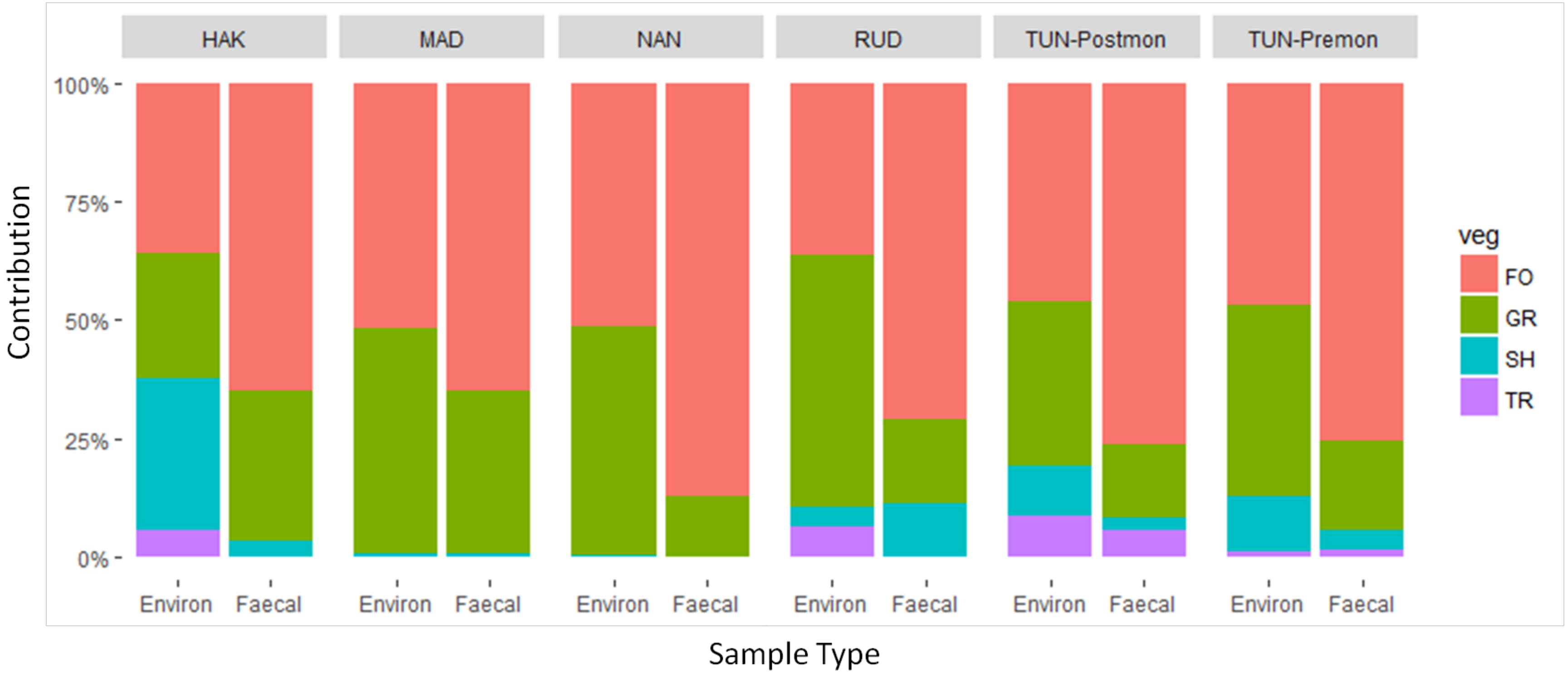

References :

Bannikov, A. G. (1954) Mlekopitayushchie Mongol'-skoi Narodnoi Respublik [Mammals of the Mongolian People's Republic]. *Academy of Sciences of the USSR,* Nauka Press, Moscow. 669 pp (In Russian).

Borisova, N. G., Starkov, A. I., Sazonov, G. I., & Rudneva, L. V. (2001) On the ecology of the daurian pika in south-western Transbaikalia. *Trudy Zoologicheskogo Instituta RAN*, 288,163–179.

Dearing, M. D. (1996) Disparate determinants of summer and winter diet selection of a generalist herbivore, *Ochotona princeps*. *Oecologia*, *108*(3), 467–478.

Dearing, M. D. (1997). The manipulation of plant toxins by a food-hoarding herbivore, *Ochotona princeps*. *Ecology*, *78*(3), 774–781.

Elliott, C.L., (1980) Quantitative analysis of pika (*Ochotona princeps*) hay piles in central Idaho. *Northwest Science*, 54, 207–209.

Fan, N., Jing, Z.& Zhang, D.(1995) Studies on the food resource niches of plateau pika and Daurian pika. *Acta Theriologica Sinica* 15, 36–40. (In Chinese with English summary)

Ge, D., Zhang, Z., Xia, L., Zhang, Q., Ma, Y., & Yang, Q. (2012) Did the expansion of C4 plants drive extinction and massive range contraction of micromammals? Inferences from food preference and historical biogeography of pikas. *Palaeogeography, Palaeoclimatology, Palaeoecology*, *326*, 160–171.

Gliwicz, J., Pagacz, S., & Witczuk, J. (2006). Strategy of food plant selection in the Siberian northern pika, *Ochotona hyperborea*. *Arctic, Antarctic, and Alpine Research*, *38*(1), 54–59.

Gravatt, D. A., & Martin, C. E. (1992) Comparative ecophysiology of five species of Sedum (Crassulaceae) under well-watered and drought-stressed conditions. *Oecologia*, *92*(4), 532–541.

Jiang, Z., & Xia, W. (1985) Utilization of the food resources by plateau pika. *Acta Theriologica Sinica*, *5*(4), 251–262.

Johnson, D. R. (1967) Diet and reproduction of Colorado pikas. *Journal of Mammalogy*, 48(2), 311–315.

Lay, D. M., (1967). A study of the mammals of Iran, resulting from the Street Expedition of 1962-63. *Fieldiana Zoology* 54, 1–282.

Liu, W., Zhang, Y., Wang, X., Zhao, J., Xu, Q., & Zhou, L. (2008) Food selection by plateau pikas in different habitats during plant growing season. *Acta Theriologica Sinica*, *28*(4), 358-366. (In Chinese with English summary)

Liu, W., Zhang, Y., Wang, X., Zhao, J., Xu, Q., & Zhou, L. (2009a) Food selection pattern for plateau pika in winter. *Acta Theriologica Sinica*, *29*(1), 12–19. (In Chinese with English summary)

Liu, W., Zhang, Y., Wang, X., Zhao, J. Z., Xu, Q. M., & Zhou, L. (2009b) The relationship of the harvesting behavior of plateau pikas with the plant community. *Acta Theriologica Sinica*, 29(1), 40–49. (In Chinese with English summary)

Liu, W., Zhang, Y., Wang, X., Zhao, J. Z., Xu, Q. M. & Zhou, L. (2009) Cachingselection by plateau pika and its biological significance. *Acta Theriologica Sinica*, 29, 152–159.

Millar, J. S. & Zwickel, F. C., (1972) Characteristics and ecological significance of hay piles of pikas. *Mammalia* 36(4), 657–667

Ognev, S.I. (1940) Zveri SSR i prilezhashchikh stran. Gryzunny. (Zveri vostochnoi Evropy i severnoi Azii) Mammals of the USSR and adjacent countries: Rodents (Mammals of eastern Europe and northern Asia). *Academiya Nauk SSSR*, 4, 1–615 (In Russian).

Osborne, C.P., Salomaa, A., Kluyver, T.A., Visser, V., Kellogg, E.A., Morrone, O., Vorontsova, M.S., Clayton, W.D. &Simpson, D.A. (2014) A global database of C_4 photosynthesis in grasses. *New Phytologist*, 204(3), 441–446.doi: 10.1111/nph.12942

Rausch, R. L. (1961) Notes on the collared pika, Ochotona collaris (Nelson), in Alaska. *The Murrelet*, *42*(2), 22–24.

Sage, R. F. (2016) Photosynthesis: Mining grasses for a better Rubisco. *Nature Plants*, *2*(12), 16192.

Su, J., Lian, X., Zhang, T., Cui, Q. & Liu, J. (2004) Hay-pile caches as winter food by Gansu pikas and its biological significance. *Acta Theriologica Sinica* 24, 23–29. (In Chinese with English Summary)

Sun Y.D., Du Y.N., Wang Z.C., Cai F.K. & Qiu B.H. (2008) Analysis of the species of storing hay by *Ochotona alpine* in Korean pine-broadleaf forest belt. *Journal of Agriculture Science Yanbian University* 30, 22–25. (In Chinese with English Summary)

Wang, X., Liu, J., Liu, W., & Ji, L. (1992) Studies on the nutritional ecology of herbivorous small mammals; patterns of food selection and food quality for plateau pikas, *Ochotona curzoniae*. *Acta Theriologica Sinica* 12, 183–192. (In Chinese with English summary)

Ye, R. R. (2006) An analysis of nutrient composition of Plateau pika diet. *Acta Laboratorium Animalis Scientia Sinica* 4, 77–81. (In Chinese with English summary)
